# Enhanced Antimicrobial and Anticancer Activity of Silver and Gold Nanoparticles Synthesised Using *Sargassum incisifolium* Aqueous Extracts

**DOI:** 10.3390/molecules21121633

**Published:** 2016-12-02

**Authors:** Mokone Mmola, Marilize Le Roes-Hill, Kim Durrell, John J. Bolton, Nicole Sibuyi, Mervin E. Meyer, Denzil R. Beukes, Edith Antunes

**Affiliations:** 1 School of Pharmacy, University of the Western Cape, Bellville 7535, South Africa; mcozeemm@gmail.com; 2 Department of Biotechnology, University of the Western Cape, Bellville 7535, South Africa; nsibuyi@uwc.ac.za (N.S.); memeyer@uwc.ac.za (M.E.M.); 3 Biocatalysis and Technical Biology Research Group, Institute of Biomedical and Microbial Biotechnology, Cape Peninsula University of Technology, Bellville 7535, South Africa; LeRoesM@cput.ac.za (M.L.R.-H.); DurrellKa@gmail.com (K.D.); 4 Department of Biological Sciences, University of Cape Town, Rondebosch 7701, South Africa; john.bolton@uct.ac.za; 5 Department of Chemistry, University of the Western Cape, Bellville 7535, South Africa; Ebeukes@uwc.ac.za

**Keywords:** silver nanoparticles, gold nanoparticles, green synthesis, fucoidans, *Sargassum incisifolium*, antibacterial activity, cytotoxicity, total phenolic content

## Abstract

A detailed, methodical approach was used to synthesise silver and gold nanoparticles using two differently prepared aqueous extracts of the brown algae *Sargassum incisifolium*. The efficiency of the extracts in producing nanoparticles were compared to commercially available brown algal fucoidans, a major constituent of brown algal aqueous extracts. The nanoparticles were characterised using TEM, XRD and UV/Vis spectroscopy and zeta potential measurements. The rate of nanoparticle formation was assessed using UV/Vis spectroscopy and related to the size, shape and morphology of the nanoparticles as revealed by TEM. The antioxidant, reducing power and total polyphenolic contents of the aqueous extracts and fucoidans were determined, revealing that the aqueous extracts with the highest contents produced smaller, spherical, more monodisperse nanoparticles at a faster rate. The nanoparticles were assessed against two gram-negative bacteria, two gram-positive bacteria and one yeast strain. In contrast to the literature, the silver nanoparticles produced using the aqueous extracts were particularly toxic to Gram-negative bacteria, while the gold nanoparticles lacked activity. The cytotoxic activity of the nanoparticles was also evaluated against cancerous (HT-29, MCF-7) and non-cancerous (MCF-12a) cell lines. The silver nanoparticles displayed selectivity, since the MCF-12a cell line was found to be resistant to the nanoparticles, while the cancerous HT-29 cell line was found to be sensitive (10% viability). The gold nanoparticles displayed negligible toxicity.

## 1. Introduction

The novel characteristics of nanomaterials compared to their bulk materials have led to intensive research in the field of nanoscience for applications in many sectors including catalysis, drug delivery, diagnostics, transport, energy, cosmetics and the development of new drugs [1,2]. The increase in surface area as the size of the material decreases, together with the quantum confinement effects which dominate at the nano-level, result in extraordinary mechanical, electrical, conductivity, magnetic and optical properties [3,4].

The emergence of multiple drug resistant microorganisms is a burden to the economic and public health sectors worldwide [5]. Inappropriate use of antibiotics, for example, has enabled microorganisms to develop mutations leading to increased resistance to antibiotics, resulting in costly, prolonged periods of hospitalization and increased mortality rates [6,7,8]. A cost-effective, broad spectrum antibiotic is thus sought and the use of nanomaterials in the development of an effective treatment against drug resistant bacteria offers a particularly attractive incentive [9]. Silver, in its bulk form, has long been known to kill most microorganisms effectively [10]. For this reason, silver nanoparticles (AgNPs), with their attractive physicochemical properties, size and surface plasmon resonance (SPR) behaviour, have captured the attention of many researchers leading to intensive research into a wide range of applications including the chemical, biological and physical sectors [11]. AgNPs have been found to possess anti-fungal, anti-bacterial and anti-inflammatory activities [4] and they are widely employed in cosmetics, wound dressings, medical implants, food packaging, water disinfection, as well as with hospital equipment to avoid infection [12,13].

The increasing interest in the application of these nanoparticles together with an increasing concern for the environment, has necessitated the need to develop mild, energy efficient and environmentally friendly synthetic methods for the production of nanoparticles. Thus, in recent years the use of plant extracts and natural products for the synthesis of nanoparticles has received a great deal of attention [14,15,16,17,18]. *Aloe vera* extracts were used by Chadran et al. to synthesise AgNPs [19], while Leela and Vivekanandan compared the capabilities of different extracts of *Helianthus annus* (sunflower) which exhibited an exceptional capacity to effect the reduction of Ag^+^ to Ag(0) [20]. Furthermore, Gnanojobitha et al. used extracts from the fruit of *Vitis vinifera* to synthesise AgNPs and evaluated their antimicrobial efficacy against *Bacillus subtilis* and *Klebsiella planticola* (Gram-positive and Gram-negative bacteria, respectively) [21]. The AgNPs were found to be active against Gram-positive microorganisms to a greater extent than the Gram-negative bacteria. Although several studies have been reported on the synthesis of AgNPs using plant extracts, these have focused only on nanoparticle formation and the experimental details for the conditions employed in the synthesis are vague. Several studies involving the biosynthesis of metallic nanoparticles using marine algae have also been reported. A few examples include AgNPs which have been synthesised using a green seaweed, *Codium capitatum* (Chlorophyta) [14] and a red seaweed, *Gelidiella acerosa* (Rhodophyta) [22]. The AgNPs in the latter study were evaluated for their antifungal activity and the greatest activity was observed against *Mucor indicus* and *Trichoderma reesei*, while moderate activity was recorded when the AgNPs were tested against *Fusarium dimerum* and *Humicola insolens* [22].

Gold nanoparticles have found use in catalysis, nonlinear optics, nanoelectronics, gene expression, and disease diagnosis [23]. Due to their biocompatibility, the medical applications for AuNPs include drug delivery, tissue/tumour imaging, photothermal therapy and the immunochromatographic identification of pathogens in clinical specimens [24]. Studies have also been conducted on the use of plant extracts to synthesise gold nanoparticles, including studies by Tripathy et al. [25] who used the leaf extracts of *Ficus benghalensis* (the banyan tree) and Sathishkumar et al. [26] who synthesised AuNPs from *Couroupita guianensis*—also known as the cannonball tree (Aubl.). A few studies have been reported on the use of marine algae to synthesise AuNPs such as *Sargassum wightii* [27] and *Stoechospermum marginatum* (Kützing) [24], where the synthesised NPs were tested for antibacterial activity against a panel of infectious microorganisms. In the latter study, Rajathi et al. demonstrated that the AuNPs were active against Gram-negative bacteria than Gram-positive bacteria [24].

Seaweeds are known for their high metal uptake capacity, their ready availability and their macroscopic structures; their large size making the biomass readily available [14]. Seaweeds have long been used for their medicinal properties (including anti-bacterial and anti-fungal activities) and they contain anti-oxidants which can act against degenerative diseases [28]. Marine brown algae contain polysaccharides which have not been found in terrestrial plants; these are called “fucoidans” according to IUPAC rules [29]. Fucoidans are thought to play a role in the synthesis of NPs due to their sulphonated side chains [30] and several commercially available fucoidans were thus used as controls in this study. In addition to the fucose and sulphate moieties, fucoidans may be composed of other monosaccharides (mannose, galactose, glucose, and xylose), uronic acids, acetyl groups and proteins [31]. Several complex phlorotannins displaying potent antioxidant activities [32] have also been isolated from brown algae, which may be very useful in the reduction of the metal salts employed to form nanoparticles. Reactive oxygen species (ROS) such as superoxide (O_2_^−^), hydroxyl (OH^−^), peroxyl (ROO^−^) and nitric oxide radicals (NO^−^) cause oxidative stress in cells and antioxidants are known to play an important role in protecting these cells. The antioxidant activity exhibited by plants and marine algae have been evaluated in numerous reports [33,34,35,36]. Polysaccharides from seaweeds such as *Ulva lactuca* (a green seaweed), *Sargassum crassifolium* and *Turbinaria ornata* (brown seaweeds) and *Digenea simplex* (a red seaweed) were evaluated by several researchers, and the highest activity was observed for the polysaccharides isolated from the brown seaweeds [37,38]. To the best of our knowledge, the antioxidant activity of *Sargassum incisifolium* has not been investigated previously.

In this study, we focus our attention on the use of aqueous extracts of the seaweed *Sargassum incisifolium*, an indigenous South African species [39,40], in the green synthesis of silver and gold nanoparticles. The seaweed aqueous extracts and commercially available pure fucoidans from other brown alga were evaluated for their antioxidant, antimicrobial and cytotoxic activities. The AgNPs and AuNPs synthesised were subsequently assessed for their potential as antimicrobial and cytotoxic agents.

## 2. Results and Discussion

### 2.1. Characterisation of the Sargassum incisifolium Aqueous Extracts and Fucoidans Used in the Synthesis of the NPs

#### 2.1.1. Sample Preparation

The aqueous extracts (AE) were prepared using two methods and the extracts are referred to as AC and AR extracts (where AC is the aqueous extract obtained after organic extraction and AR is the aqueous extract obtained as is from the seaweed). Both extracts were brown in colour which may be attributed to the presence of polyphenols which have been previously isolated from brown algae [41], in contrast to the fucoidan solutions which were colourless (Figure S1). These extracts were further characterised by UV/Vis (Figure S2), 2D NMR and FT-IR spectroscopies (Figures S3–S7) and is discussed in the Supplementary Information.

#### 2.1.2. Determination of the Antioxidant Activity, Polyphenolic Content and Total Reducing Power of the Aqueous Extracts of *S. incisifolium* and Fucoidans

We hypothesised that antioxidant compounds in the aqueous extract of *S. incisifolium* would be capable of reducing Ag^+^ and Au^3+^ to Ag(0) and Au(0). This part of the study was therefore carried out to give insight into (a) the polyphenolic content of the reagents used and their reducing power (and therefore their ability to form nanoparticles) and (b) their antioxidant activity. The polyphenolic content, total reducing power and antioxidant activity of the two aqueous extracts of *S. incisifolium* and pure fucoidans (Fv, Mp, Up) were evaluated according to Topiwala et al. [36] and the results are presented in Table 1.

The total phenolic contents of the samples were recorded as gallic acid equivalents (GAE) in µg/mg of dried seaweed. Prior extraction of the seaweed with organic solvents rendered the AC extract (which is a darker brown in colour) a higher phenolic content and reducing power compared to the AR extract. The pure fucoidans possessed significantly lower phenolic contents than the aqueous extracts with Fv, at 1 µg/mg GAE, having the lowest content (Table 1). The total phenolic content was observed in the following order: AC > AR > Mp > Up > Fv (with *p* < 0.05).

The total reducing power of these samples was also assessed and a similar trend observed. In this assay, the total reducing power of the samples depends on the capability of the antioxidants in the samples to reduce Fe^3+^ (and give an indication of the ability of the samples to reduce the Ag and Au salts to form nanoparticles). The results obtained (Table 1) are presented as ascorbic acid equivalents (AAE) in µg/mg of dried seaweed and pure fucoidans. The AC extract boasted a higher total reducing power content (at 95 µg/mg AAE of dried seaweed) compared to the AR sample and pure fucoidans (Table 1). The pure fucoidans exhibited much lower values, with the lowest values recorded for the Fv fucoidan (at 10 µg/mg AAE). The following order was observed for the total reducing power: AC > AR > Mp > Up > Fv.

**Figure 1:**
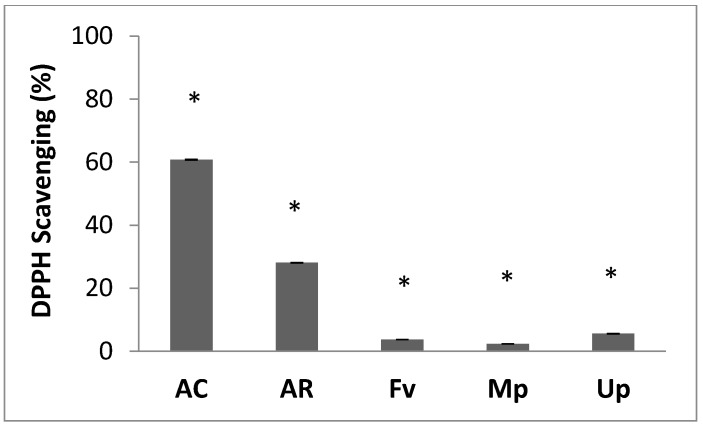
The DPPH radical scavenging power of the *S. incisifolium* aqueous extracts (AC and AR), and fucoidans from *F. vesiculosus* (Fv), *M. pyrifera* (Mp) and *U. pinnatifida* (Up). * *p* < 0.05. AC: Aqueous extract subjected to organic solvent partitioning. AR: simple aqueous extraction.

The trends observed above were echoed in the radical scavenging assays conducted. The DPPH scavenging method is dependent on the reduction of the DPPH to a more stable form (DPPH-H) by the antioxidants [42]. The AC extract once again exhibited a higher radical scavenging power (60% DPPH scavenging) compared to the AR extract (28% DPPH scavenging, Figure 1). The % radical scavenging ability of the fucoidans was again found to be low, with the lowest value recorded for the Mp fucoidan. The following order was observed with the radical scavenging ability: AC > AR > Up > Fv > Mp.

The aqueous extract samples as prepared would contain quantitatively less fucoidan than the commercial fucoidan samples, and these data therefore shows that the fucoidans are poor antioxidants and it is most likely the phlorotannins (in the aqueous extracts) that will be important in reducing the metal salts.

### 2.2. Synthesis and Characterisation of the AgNPs and AuNPs

#### 2.2.1. Preparation of AgNPs Using Sodium Borohydride and *S. incisifolium* Aqueous Extracts

**Figure 2:**
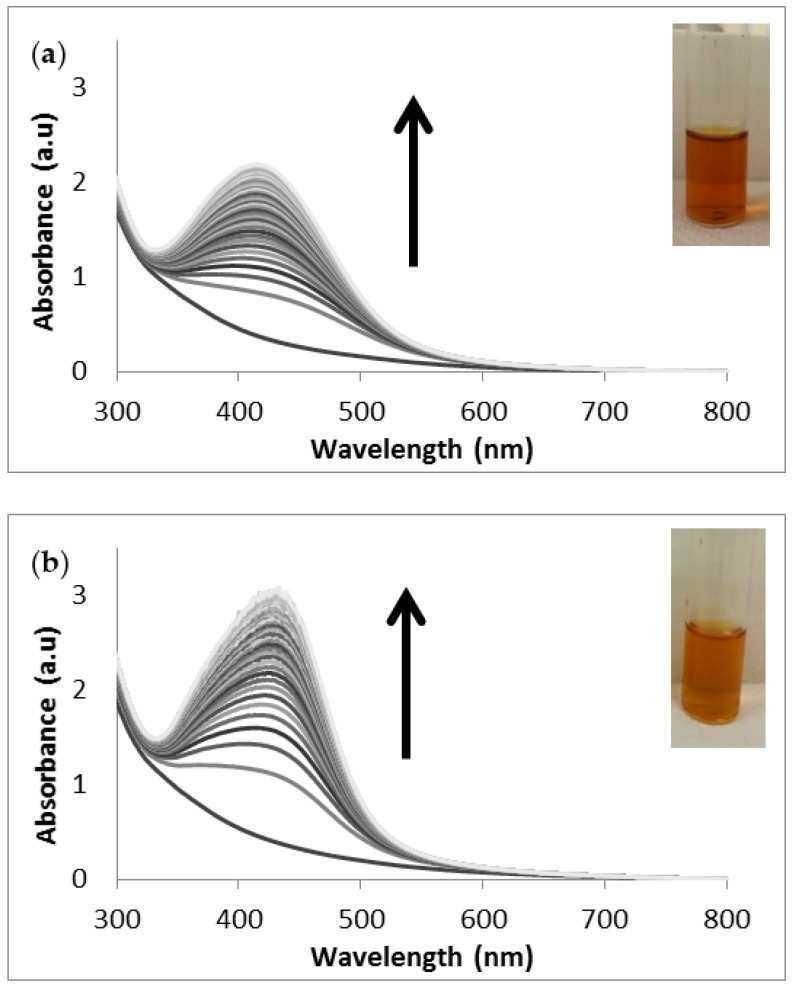
UV-Vis absorption spectra of (**a**) the AC-AgNPs and (**b**) the AR-AgNPs from time 0 to 18 h. Inset: Photograph of the colour of the solution after 18 h (in water). AC: Aqueous extract subjected to organic solvent partitioning. AR: simple aqueous extraction.

**Figure 3:**
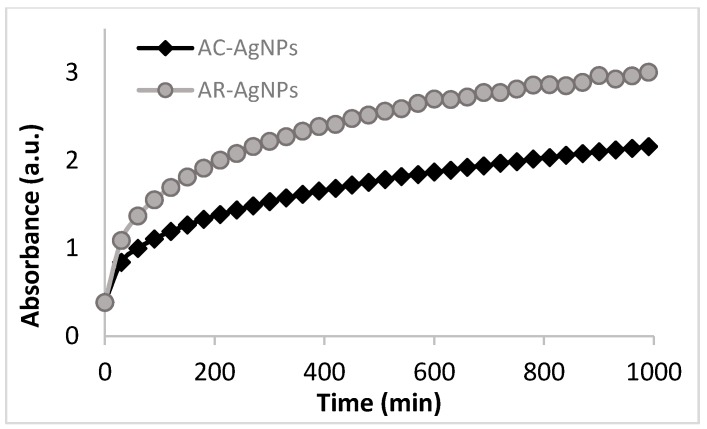
Change in absorbance with time during AC-AgNP and AR-AgNP formation as observed at the λ_max_ at 413 nm and 433 nm, respectively (in water). AC: Aqueous extract subjected to organic solvent partitioning. AR: simple aqueous extraction.

The synthesis of AgNPs using sodium borohydride (SB-AgNPs) was used as a control reaction since it is typically reported in the literature and used as a comparison with the ability of the fucoidans and aqueous extracts to produce AgNPs. The NPs formed easily with NaBH_4_ (Figure S8) and the aqueous extracts (Figure 2 and Figure 3), with the UV/Vis absorption spectra revealing the characteristic SPR bands [17] for the AgNPs at 397 nm and ~425 nm for the NaBH_4_ and aqueous extracts, respectively.

Most studies describe the use of liquid extracts from plants or seaweeds to synthesise nanoparticles without determining the concentration of extracts required for the synthesis. In this study, freeze-dried extracts were used to synthesise nanoparticles to standardise the procedures. The synthesis of the AgNPs using these *S. incisifolium* aqueous extracts (both AC and AR) were found to proceed easily at room temperature and pressure conditions (i.e., using energy efficient and safe methods). Confirmation of the presence of AgNPs in the solution was obtained by observing the presence of the SPR band in the 410–440 nm range (Figure 2) in the UV-Vis spectra. Interestingly, the SPR bands of the NPs produced with the AR extract (at 433 nm) revealed a red shift of 20 nm compared to those produced with the AC extract (at 413 nm). The formation of the nanoparticles was found to occur within 30 min, though the speed of the reaction appeared to slow down after 120 min for both extracts (Figure 3). The reaction was allowed to proceed for a further 18 h to ensure completion (as evidenced by the plateau in the absorption λ_max_ observed in Figure 3).

#### 2.2.2. Preparation of AgNPs Using Commercially Available Fucoidans

**Figure 4:**
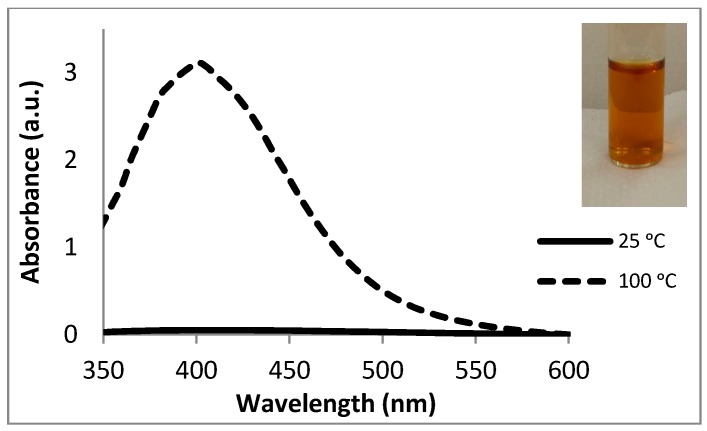
UV-Vis absorption spectra (in water) for the Fv-AgNPs after 18 h at room temperature (in water), and after 15 min at 100 °C. Inset: Photograph of the colour of the solution after 15 min (in water).

AgNPs were synthesised in this study using commercially available fucoidans from brown seaweeds viz. *F. vesiculosus* (Fv), *M. pyrifera* (Mp) and *U. pinnatifida* (Up), to assess whether the polysaccharides can reduce the Ag salt to form the NPs (in addition to capping the NPs). The UV-Vis spectra obtained for the NPs are shown in Figure 4 and Figure S9. As shown in the spectra, AgNP formation did not occur at room temperature, suggesting that the pure fucoidans are not solely responsible for the reduction of the silver ions at room temperature to form AgNPs. Increasing the reaction temperature to 100 °C resulted in the formation of the AgNPs to some extent, with reaction times varying from 15 min to 1 h.

The Fv fucoidans showed the greatest potential for AgNP formation with the Up fucoidan the least. Nevertheless, these results do not preclude the fucoidans from acting as good capping agents, ensuring the biocompatibility of the NPs produced. It is thus apparent that the fucoidans could only produce NPs at elevated temperatures, while the aqueous extracts of *S. incisifolium* could produce NPs easily. It is therefore clear that other secondary metabolites (such as phlorotannins which have also been isolated from brown algae) are responsible for reducing the Ag ions to form the NPs. The results obtained from the total phenolic contents for each of the aqueous extracts certainly points to the likelihood of the involvement of phlorotannins. It is also likely that the increased temperatures may break down the fucoidans into sugars that can reduce the metal salt to produce the AgNPs.

#### 2.2.3. Preparation of AuNPs Using Sodium Citrate and *S. incisifolium* Aqueous Extracts

Gold nanoparticles (AuNPs) were also synthesised in this study as the characteristic pink SPR band of AuNPs (at ~530 nm) is much easier to visualise than the AgNP SPR band. These AuNPs also used as a control in the antimicrobial studies since AuNPs are not expected to possess antimicrobial properties, unlike AgNPs. Furthermore, these NPs are also likely to indicate whether the NPs are toxic to normal cell lines i.e., they may give some indication as to the safety and efficacy of these NPs for use in humans.

The synthesis of sodium citrate capped AuNPs (labelled SC-AuNPs) was accomplished using well-established literature methods [43]. The NPs produced using sodium citrate were only successfully synthesised at higher temperatures (i.e., 90 °C), with the formation of the nanoparticles apparent after 5 min (Figure S10).

**Figure 5:**
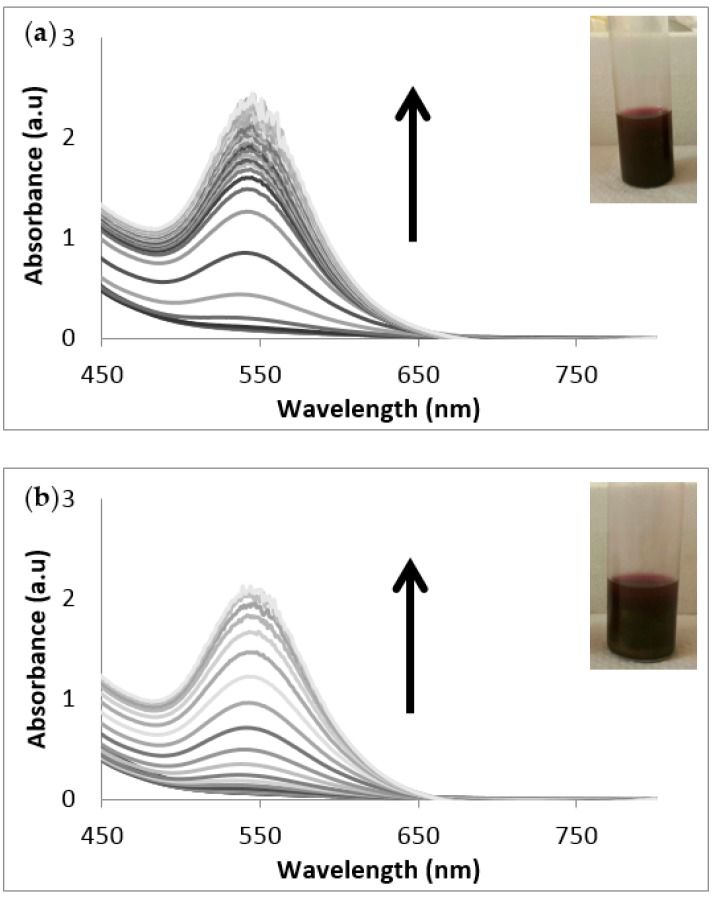
UV-Vis absorption spectra of (**a**) AC-AuNPs and (**b**) AR-AuNPs formed after 5 h at room temperature (in water). Inset: Photograph of the colour of the solution after 5 h. AC: Aqueous extract subjected to organic solvent partitioning. AR: simple aqueous extraction.

Similarly, AuNPs were also synthesised using the AC and AR extracts (Figure 5). The formation of the AuNPs was easily observed by the change of colour of the solution from yellow to dark purple (inset Figure 5).

**Figure 6:**
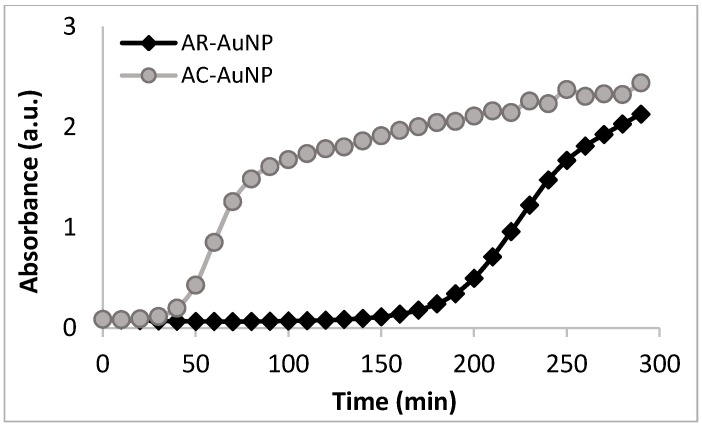
Change in absorbance with time during the syntheses of both the AC-AuNPs and AR-AuNPs at the λ_max_ 545 and 544 nm respectively (in water). AC: Aqueous extract subjected to organic solvent partitioning. AR: simple aqueous extraction.

Interestingly, a difference in reaction rates was observed for these extracts, with NP formation appearing to slow down after 50 min for the AC extract, and after 95 min with the AR extract (Figure 6). The NP formation rate was therefore slower for the AR extracts, and this difference may once again be attributed to the difference in polyphenolic content, reducing power and antioxidant potential of the two aqueous extracts.

An attempt to produce AuNPs with the commercial fucoidans was unsuccessful at both room and elevated temperatures (Figure S11). It is thus apparent that fucoidans do not play a role in the reduction of the metal ions to form the NPs. These results contrast with the results obtained by Soisuwan et al. [44] who could synthesise AuNPs using pure fucoidans from the brown seaweeds *Cladosiphon okamuranus* and *Kjellmaniella crassifolia* at room temperature as well as at 80 °C. The latter may be attributed to the fact that *Kjellmaniella crassifolia* is a very different species of brown algae compared to the *S. incisifolium* used in this study, although sulfated polysaccharides have also been isolated from this seaweed [45]. However, this would depend on the purity of the fucoidan samples and whether they contain any polyphenol impurities.

### 2.3. Characterization of the Synthesised Nanoparticles

#### 2.3.1. Transmission Electron Microscopy (TEM) and Energy Dispersive X-ray (EDX) Analyses

**Figure 7:**
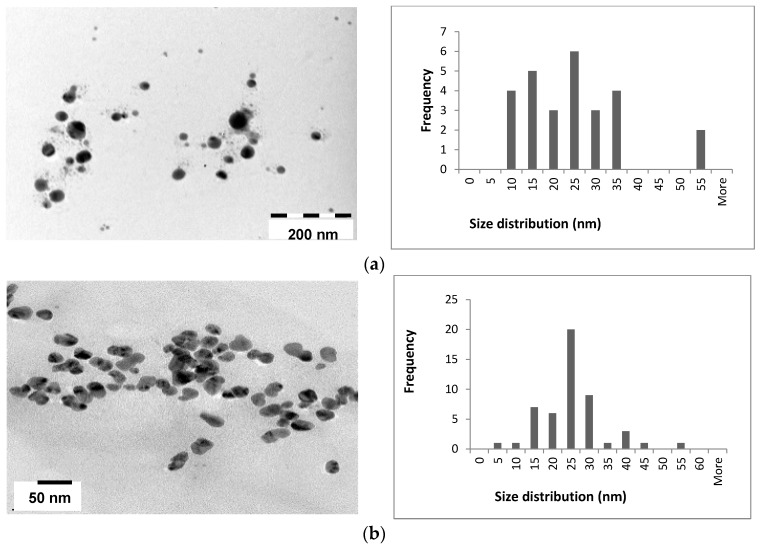

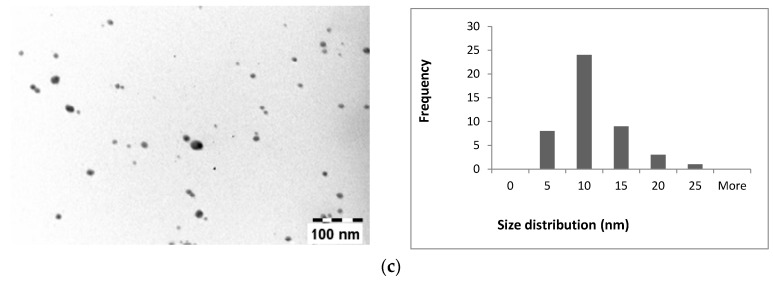
TEM images and NP size distributions obtained for (**a**) AC-AgNPs; (**b**) AR-AgNPs and (**c**) Fv-AgNPs. AC: Aqueous extract subjected to organic solvent partitioning. AR: simple aqueous extraction.

**Figure 8:**
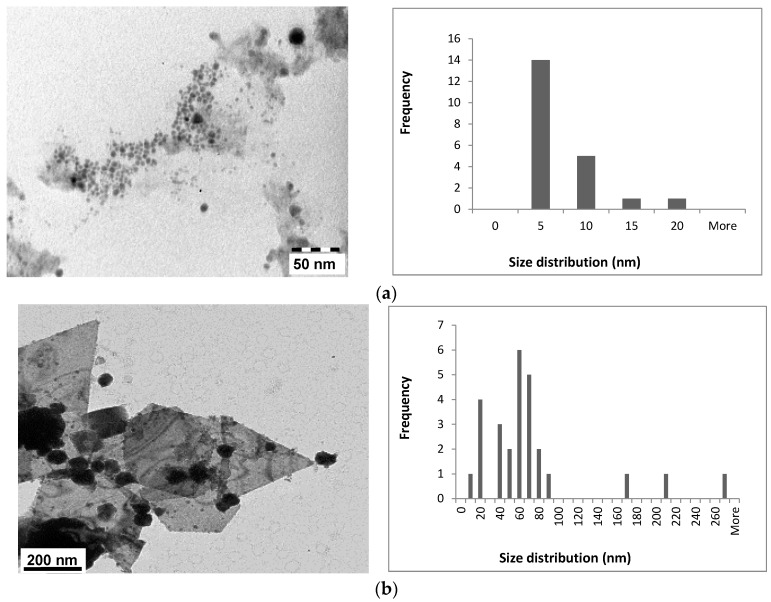
TEM images and NP size distributions obtained for (**a**) AC-AuNPs and (**b**) AR-AuNPs. AC: Aqueous extract subjected to organic solvent partitioning. AR: simple aqueous extraction.

The TEM results obtained for the Ag and Au NPs synthesised are shown in Figure 7 and Figure 8 (and Figures S12 and S14). The mostly spherical AgNPs are well dispersed and have an average size of 20 nm (Table 2), although a large size range was obtained for the AgNPs produced with NaBH_4_ (Figure S11). The TEM images for the NPs synthesised using the AC and AR extracts (Figure 7) show a uniform size range for the AR-AgNPs, although the NPs are not as well dispersed as those obtained with the AC extracts (Figure 7a). The Fv and Mp fucoidans produced NPs which were spherical and well dispersed, however, a large size range was obtained. The Fv-AgNPs were found to be the smallest at 8.7 nm (Figure 7c). If the fucoidan structure is highly branched, they are thought to bind to several NPs simultaneously, especially if there are sulphonated functional groups on the side chains resulting in larger, agglomerated NPs [44]. To the best of our knowledge, fucoidans from *S. incisifolium* have not been characterised and its structure remains unknown.

Spherically shaped AuNPs with an average size of 12.38 nm (Table 3) as revealed by TEM were obtained for the sodium citrate capped NPs (Figure S13). The TEM images obtained for the NPs produced with aqueous extracts, revealed well-dispersed NPs with a variety of shapes, including triangles, spheres and hexagons obtained for the AR-AuNPs which were found to be much larger in size (up to 200 nm, Figure 8b). The images for the AC NPs revealed only spherical NPs which averaged 5 nm in size (Figure 8a). Interestingly, the rate of NP formation is faster with the AC extracts, and smaller, monodisperse NPs were produced compared to the NPs produced with the AR extracts.

Elemental analyses of the nanoparticles were accomplished using Energy Dispersive X-ray (EDX) Spectroscopy, which was subsequently acquired with the TEM images. Elemental Ag and Au were detected in all samples as expected (Figures S13 and S15).

#### 2.3.2. Characterization of the Synthesised Nanoparticles Using Dynamic Light Scattering (DLS) and Zeta Potential Measurements

Using a Zetasizer, the size of the nanoparticles was also determined using DLS and the data acquired is given in Table 2 and Table 3. The size of the NPs as determined by TEM are much smaller compared to the DLS measurements, which is expected as DLS measures the hydrodynamic (d_H_) radius of the nanoparticles suspended in water together with any coating material on the surface of the nanoparticle [46,47]. The difference in size for the TEM and DLS data can therefore be assumed to be due to the capping agent present on the NP surface, i.e., the fucoidans and/or phlorotannins expected to be present in the extracts.

The polydispersity index (PdI) of the NPs may also be determined using the Zetasizer and the data is listed in Table 2 and Table 3. A PdI index of less than 0.1 represents monodispersed NPs, while an index of between 0.1 ≤ 0.2 indicates a narrow size distribution, while an index between 0.2 ≤ 0.5 indicates a broad size distribution for the sample [48]. From the results obtained, the NPs all show a broad size distribution, since the PdI index ranges from 0.264 to 0.568, except for SC-AuNPs which have a narrow size distribution (0.158), as indicated by the TEM data.

The Zeta potential measurements obtained for the NPs are listed in Table 2 and Table 3 for both the AgNPs and AuNPs. From these data, the NPs all have a negative potential surface charge as expected given the structure of the fucoidans. The AR-AgNPs possessed the largest negative charge (at −40.0 mV) for the samples synthesised using the aqueous extracts, while the NPs synthesised using the fucoidans showed that the Fv-AgNP sample exhibited the largest negative charge at −44.1 mV. The sodium citrate capped AuNPs, however, displayed the largest negative charge of all, implying that these NPs were the most stable [49], while the AgNP synthesised using NaBH_4_ had the lowest negative potential (Table 2 and Table 3). All the nanoparticles synthesised exhibited zeta potentials within the cut-off range, i.e., NPs with zeta potential values greater than +30 mV or less than −30 mV are associated with inherent stability [49].

#### 2.3.3. Characterization of Synthesised Nanoparticles Using Inductively Coupled Plasma—Atomic Emission Spectroscopy (ICP-AES)

ICP-AES was used to determine the extent of reaction completion in formation of the NPs. The results are shown in Table 2 and Table 3. The samples were subjected to centrifugation and the concentration of the metal ions (in ppm) remaining in the supernatant were taken as an indication of the amount of unreacted metal salt. 

The percentage of Ag ions in the supernatant of the AgNPs reaction solution was found to be 30%–39%, with the Fv-AgNPs and AC-AgNPs containing the highest percentage (Table 2), indicating that the reaction was still not complete after 18 h for the AgNPs synthesised with the AC extract at room temperature and after 15 min for Fv-AgNPs at 100 °C. Syntheses of the AuNPs on the other hand was more successful with only 14% metal content present in the supernatant (Table 3).

#### 2.3.4. Characterization of the Synthesised Nanoparticles Using Powder X-ray Diffraction (XRD)

**Figure 9:**
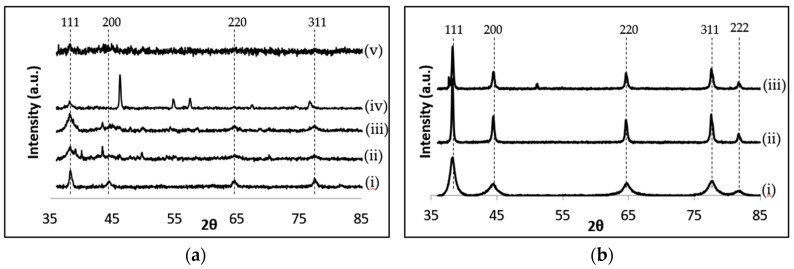
XRD patterns obtained for (**a**) the AgNPs synthesised using (i) sodium borohydride; (ii) AC extracts; (iii) AR extracts; (iv) Mp fucoidan and (v) Fv fucoidans; and (**b**) the AuNPs synthesized using (i) sodium citrate; (ii) AC extracts and (iii) AR extracts. AC: Aqueous extract subjected to organic solvent partitioning. AR: simple aqueous extraction.

The synthesised NPs were characterized using powder XRD and the patterns are shown in Figure 9. The XRD reflections obtained for the AgNPs (Figure 9a) observed at 2θ = 38.1°, 44.2°, 64.3° and 77.6° can be attributed to the (111), (200), (220), and (311) crystalline planes of the face-centred-cubic (fcc) crystalline structure of metallic silver, respectively (as per JCPDS file no. 00-004-0783). The spectra revealed that the AgNPs are not the only components present since additional reflections can be seen, particularly in Figure 9a (iv), at 2θ = 46°, 55°, 57.5°, 67.7° and 77.5° which may be attributed to the presence of unreacted AgCl still present in the Mp-AgNP sample. This was later confirmed using ICP-AES analyses (Table 2). It is also clear that Fv-AgNP formation (Figure 9a (v)) was not successful, although the UV-Vis spectra revealed an SPR band and NPs were observed in the TEM images.

The AuNPs synthesised were also characterised by powder XRD and shown in Figure 9b. The patterns obtained for the AuNPs revealed broad reflections, indicating the presence of NPs. The clear reflections implied that the syntheses of these NPs were more successful than that for the AgNPs. These were indexed to 2θ = 38.2°, 44.4°, 64.6°, 77.6° and 81.7° and attributed to the (111), (200), (220), (311) and (222) crystalline planes of face-centred-cubic (fcc) crystalline structure of metallic gold (as per JCPDS file no. 00-004-0784), respectively.

From these XRD patterns the (111) facets dominate the XRD patterns of nanoparticles synthesised. Where possible, these peaks were used to calculate the both the Ag- and Au-NP sizes using the Debye-Scherrer equation (Equation (1)). The results obtained are listed in Table 2 and Table 3 and it becomes apparent that the XRD sizes do not correlate well with the sizes obtained using TEM. This may be because the TEM sizes are usually obtained from a small section of the sample grid, whereas the XRD analyses results in the determination of an average particle size for the whole sample.

### 2.4. Antimicrobial and Cytotoxicity Studies

#### 2.4.1. Antimicrobial Assays

**Figure 10:**
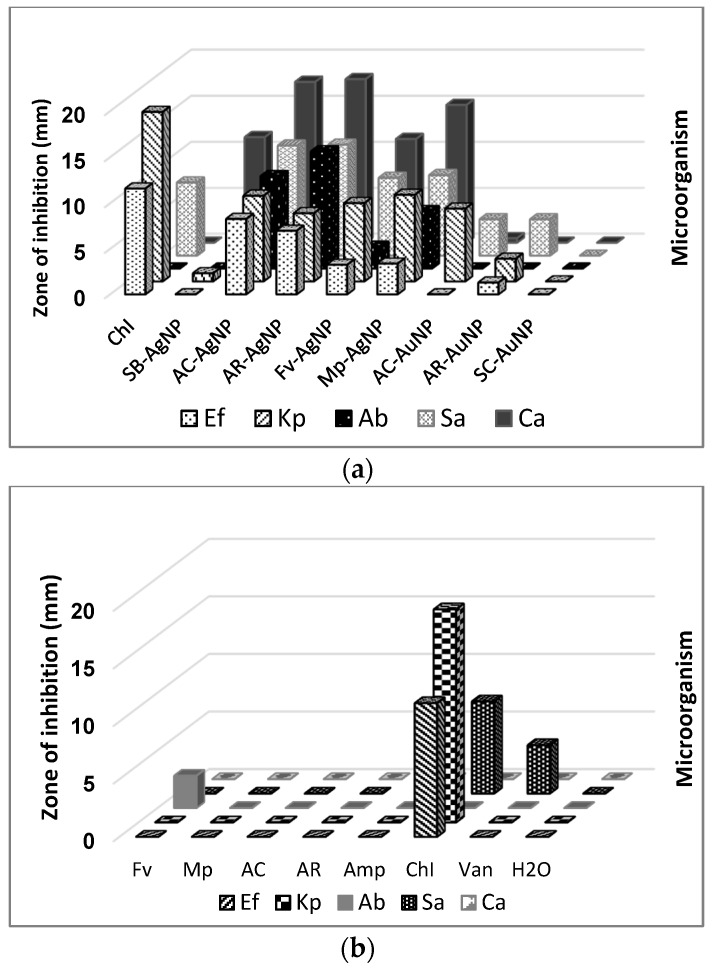
Antimicrobial activity (well-diffusion assay) against a panel of microorganisms (Ab = *A. baumannii*, Kp = *K. pneumoniae*, Ef = *E. faicalis*, Sa = *S. aureus*, Ca = *C. albicans*) for (**a**) the synthesised nanoparticles and (**b**) the aqueous extracts (AC and AR), fucoidans (Fv and Mp), and controls: Vancomycin (Van), Ampicillin (Amp), Chloramphenicol (ChI) and water (H_2_O). NP concentrations used: ~0.2 mM. [Van], [Amp] and [ChI]: 1 mg/mL. AC: Aqueous extract subjected to organic solvent partitioning. AR: simple aqueous extraction.

The antimicrobial activity of the samples was assessed against a panel of infectious microorganisms based on the well-diffusion test. The results are shown in Figure 10 and in Table S2.

The results indicate that the crude aqueous extracts of *S. incisifolium* (AC and AR), as well as the Fv and Mp fucoidans alone, showed negligible activity against the panel of microorganisms. Similarly, the NPs synthesised using sodium borohydride (SB-AgNP) as well as all AuNPs synthesised also revealed negligible antimicrobial activity (Table S2, Figure 10). The most potent activity was exhibited by the AgNPs synthesised using the aqueous extracts of *S. incisifolium* (i.e., AC-AgNPs and AR-AgNPs), showing even greater activity than the antibiotics ampicillin and vancomycin against the strains tested. It is important to note that the strains chosen are antibiotic resistant strains.

The greatest inhibition was recorded for the yeast strain, followed by the Gram-negative bacteria and finally the Gram-positive bacteria in the agar well-diffusion assay. A survey of the literature indicates that the Gram-positive bacteria are most susceptible to NPs in general. Liu et al. [50] evaluated sodium citrate capped AgNPs against *E. coli* (Gram negative) and *B. subtilis* (Gram-positive bacteria) and showed that the greatest growth inhibition was for the Gram-positive bacteria. In a separate study, Mittal et al. [51] used a plant extract from *Potentilla fulgens* to synthesise AgNPs to evaluate their antimicrobial activity, showing greater activity against the Gram-positive bacteria [51]. The difference in degree of AgNP toxicity towards Gram-positive and Gram-negative bacteria is likely to stem from the bacteria’s cell wall structural composition. The cell walls of Gram-positive bacteria consist of a thick peptidoglycan layer, techoic acid, functional protein and a single bilayer which is wrapped in lipids, while the cell walls of Gram-negative bacteria consist of a thin peptidoglycan layer embedded within two lipid bilayers and lipopolysaccharide (LPS). The LPS plays a major role in protecting the Gram-negative bacteria [52]. As such, Gram-negative bacteria are likely to be more resistant to nanoparticles than Gram-positive bacteria [50].

Surprisingly, the aqueous extracts themselves alone did not show any activity. Fucoidans have previously shown anticoagulant activity [31]. In this study, the Fv fucoidans showed some activity (2.8 mm) against *A. baumannii* only, suggesting that this fucoidan may also possess bactericidal activity (Table S2). While the Mp fucoidan itself did not show any activity against the selected microbes, the Mp-AgNP sample did show some activity against the test strains (Table S2, Figure 10). The lack of activity observed may also be ascribed to the possibility that either the bacteria may be using an antimicrobial resistance mechanism that results in resistance to the various preparations used in this study or that there is some synergistic activity between the NPs and seaweed extracts and fucoidans.

The AuNP samples were also tested against the different microorganisms as a control study, as they were not expected to possess antimicrobial activity. However, NPs synthesised from plant extracts by various research groups have shown some bactericidal activity, e.g. Park et al. [53], showed that AuNPs capped with resveratrol were toxic against *Streptococcus pneumoniae*.

#### 2.4.2. Cytotoxic Activity of the Synthesised Silver and Gold Nanoparticles

One of the major concerns in the development of AgNPs as antimicrobial agents is their potential toxicity to humans and the environment. It was therefore important to assess the cytotoxicity of the synthesised NPs. The cytotoxicity of the NPs was determined using the MTT assay where viable cells are expected to reduce the MTT dye which can then be measured by absorbance spectroscopy.

**Figure 11:**
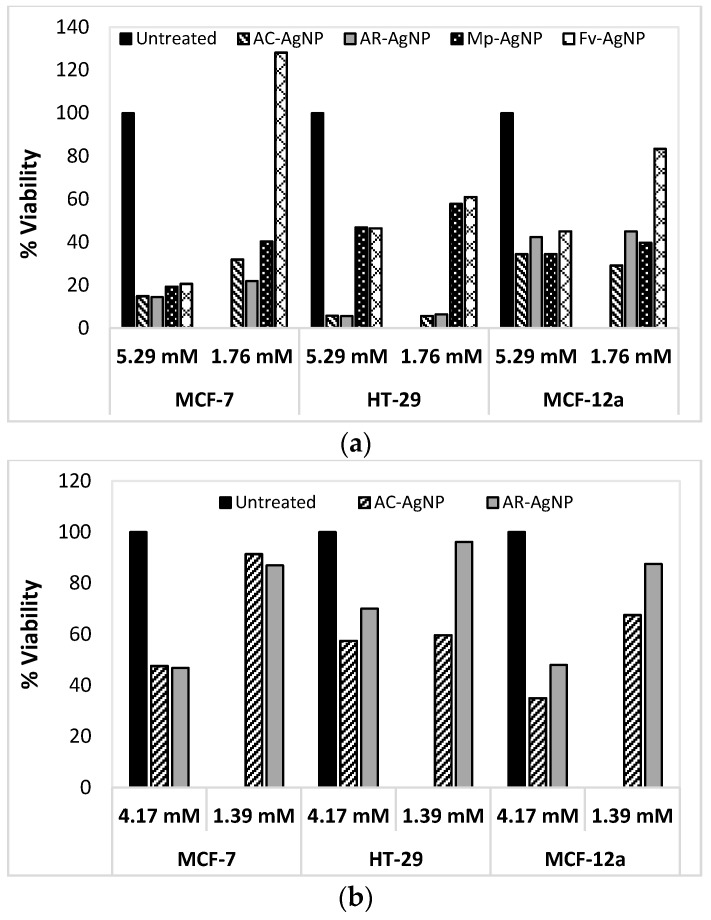
Percentage cell viability for MCF-7, HT-29 and MCF-12a cell lines after 24 h with (**a**) AgNPs and (**b**) AuNPs. AC: Aqueous extract subjected to organic solvent partitioning. AR: simple aqueous extraction.

Figure 11a shows that the AC-AgNPs and AR-AgNPs were found to be toxic to the MCF-7 cell lines at a concentration of 5.29 mM and a dose response relationship was observed when the concentration of NPs was decreased. Although the SB-AgNPs showed some activity against MCF-7, the percentage viability of these NPs was found to be above 40% (Figure S16b). AuNPs were also assessed for their cytotoxicity against the MCF-7 cell line. The AR and AC-AuNPs displayed 40% activity against the MCF-7 cell line (at 4.17 mM, Figure 11b) and a dose response relationship was again observed. The SC-AuNPs, aqueous extracts and capping agents alone showed no cytotoxic activity against the MCF-7 cell line (Figure S16b).

The toxicity of the NPs was also evaluated against human colon cancer cell line (HT-29). The AgNPs synthesised using aqueous extracts appeared to exert some toxicity towards the HT-29 cell line with the percentage viability of the cells below 10% (at 5.29 mM and 1.76 mM, Figure 11a). The fucoidan synthesised NPs appeared to be less toxic against the HT-29 cell line with their percentage viability at just above 40% (Figure 11a). A dose response relationship was observed for the HT-29 cell line, while the SB-AgNPs were found to exhibit only slight toxicity against the HT-29 cell line (Figure S17b). For comparison, the aqueous extracts and the fucoidans alone were evaluated for their activity against the HT-29cell line (Figure S17a), and only the Fv fucoidan appeared to be toxic against the HT-29 cell line (viability below 40%). As with the MCF-7 cell line, the cytotoxicity of the AuNPs against the HT-29 cell line (Figure 11b) was evaluated and the AR- and AC-AuNPs showed comparable cytotoxicity (~40%) at the highest concentration (4.17 mM). Finally, to evaluate the selectivity of the NPs synthesised for cancerous cells, a non-cancerous breast cell line, MCF-12a, was used (Figure 11). As with the cancer cell lines, a dose response relationship was also observed when the NPs were tested against MCF-12a. Although a few samples were found to exhibit some toxicity towards MCF-12a, the toxicity levels of all the NPs tested against MCF-12a are much lower compared to that of the MCF-7 and HT-29 cell lines. The results in Figure 11 show that at 5.29 mM, the Mp-AgNPs and AC-AgNPs showed some activity against MCF-12a, with percentage viabilities just below 40%, while the AR-AgNP and Fv-AgNP samples followed with percentage viabilities at just above 40% (at 5.29 mM). The aqueous extracts and fucoidans alone were also tested, where the Fv fucoidan and the AR extract showed percentage viabilities of ~40% where all other samples showed percentage viabilities of at least 60% (Figure S18). The results obtained for the MCF-12a cell line showed that only the AR-AuNPs showed some (low) activity against MCF-12a at the highest concentration (Figure 11), while the AC-AuNP did not show any activity. These preliminary results therefore indicate that these green synthesised NPs show some selectivity to cancerous cell lines.

Figure 11 compares the selectivity of the AgNPs for the cell lines tested. It appears that the MCF-7 cancer cell line is more susceptible to the AgNPs than the non-cancerous cell line (MCF-12a) which appears to be somewhat resistant to the AgNPs (Figure 11). Of the three cell lines, the HT-29 cell line appears to be the most susceptible to the AC-AgNPs and AR-AgNPs. However, the colon adenocarcinoma cell lines seemed to be less responsive to the Mp-AgNPs and Fv-AgNPs samples, unlike MCF-7 cell line. The results obtained from the studies conducted on the non-cancerous cell line, MCF-12a, suggest that AgNPs display selective cytotoxicity, since the AgNPs synthesised using *S. incisifolium* aqueous extracts are toxic towards MCF-7 cell lines, but less so towards the non-cancerous cell line, MCF-12a.

There are several mechanisms through which AgNPs may kill cancer cells with one mechanism involving the induction of oxidative stress [15,54]. The size, shape and the zeta potential of AgNPs are also known to elicit cell death through the elevation of reactive oxygen species (ROS) [54].

Even though the most active antimicrobial samples, i.e., the AC-AgNPs and AR-AgNPs, also showed the most potent cytotoxicity against the cancer cells studied, the concentrations used in the cytotoxicity studies were much higher than that used for the antimicrobial activity assays. These NPs are therefore not expected to be very toxic to humans at the much lower concentrations used for the antimicrobial assays.

## 3. Materials and Methods

The materials used in this study including sodium borohydride, sodium citrate, gold (III) chloride tryhidrate, silver nitrate, the fucoidans from *Fucus vesiculosus* (Fv), *Undaria pinnatifida* (Up) and *Macrocystis pyrifera* (Mp) seaweeds, Vancomycin (Van), Ampicillin (Amp), Chloramphenicol (ChI) were purchased from Sigma-Aldrich (St. Louis, MO, USA) and used without further purification. All solvents were redistilled before use. Milli-Q water (15.0 mΩ·cm^−1^, Millipore Merck KGaA, Billerica, MA, USA) was used in all reactions, including the extraction procedures. *S. incisifolium* was collected from Noordhoek, near Port Elizabeth on the south-east coast of South Africa and stored frozen (−20 °C) until use.

Five drug resistant strains were selected for the antimicrobial studies. These included two gram-positive strains, *Enterococcus faecalis* (ATCC 51299; a vancomycin-resistant strain) and *Staphylococcus aureus* subsp. *aureus* (ATCC 33591; a methicillin-resistant strain), two gram-negative strains, *Acinetobacter baumannii* (ATCC BAA-1605; a multi-drug resistant strain) and *Klebsiella pneumoniae* subsp. *pneumoniae* (ATCC 700603; a β-lactam resistant strain), and a yeast strain, *Candida albicans* (ATCC 24433). To adequately assess the potential of the synthesised nanoparticles as antimicrobials, it was essential to also assess their cytotoxicity against a variety of human (cancerous and non-cancerous) cell lines. The synthesised nanoparticles were evaluated against a human colon adenocarcinoma cell line (HT-29), a human breast carcinoma cell line (MCF-7) and a non-tumorigenic breast epithelial cell line (MCF-12a).

### 3.1. Synthesis of Silver Nanoparticles (AgNPs)

#### 3.1.1. AgNPs Synthesised Using NaBH_4_ (SB-AgNP)

The method used for the traditional syntheses of AgNP using NaBH_4_ was based on a method developed by Solomon et al. [55].

#### 3.1.2. AgNP Synthesis Using Commercially Available Fucoidans

The fucoidans obtained from *F. vesiculosus* (Fv), *U. pinnatifida* (Up), *M. pyrifera* (Mp) were also used to synthesise the Ag nanoparticles. Briefly, 10 mg of pure fucoidans (Fv, Mp or Up) was dissolved in 10 mL of distilled water. To this solution, 500 µL of a 0.1 M AgNO_3_ solution was added. The solution was then allowed to stir at 100 °C for 15 min for the *F. vesiculosus* fucoidin, 30 min for the *M. pyrifera* fucoidan, and 60 min for the *U. pynnatifida* fucoidan, to produce the Fv-AgNPs, Mp-AgNPs and Up-AgNPs, respectively. The reaction times chosen were dependent on the rate of NP formation.

#### 3.1.3. AgNP Synthesis Using *S. incisifolium* Aqueous Extracts

Briefly, the freeze-dried extract (2 mg) was dissolved in 10 mL of distilled water. AR extracts are aqueous extracts are simply used as is, while AC extracts are aqueous extracts firstly subjected to organic solvent partitioning. The solution was allowed to mix for 10 min after which 500 µL of a AgNO_3_ (0.1 M) solution was added and the reaction allowed to proceed for 18 h. This method was used for both the AR and AC extracts to produce the AR-AgNPs and AC-AgNPs, respectively.

### 3.2. Synthesis of Gold Nanoparticles (AuNPs)

The protocols employed in the synthesis of AuNPs were the same as that used in the synthesis of the AgNPs. Attempts to synthesise AuNPs using the commercially available fucoidans (Fv, Mp and Up) were unsuccessful and will therefore not be described here.

#### 3.2.1. Synthesis of AuNPs Using Sodium Citrate (SC-AuNPs)

The sodium citrate capped AuNPs (SC-AuNPs) were synthesised following a method used by Ojea-Jimenez et al. [43].

#### 3.2.2. Synthesis of AuNPs using *S. incisifolium* AC and AR Aqueous Extracts

The AC-AuNPs and AR-AuNPs were synthesised using the AC and AR seaweed aqueous extracts respectively, employing the same protocols described above for the AgNPs. For all synthetic procedures, the NPs synthesized were centrifuged at 10,000 rpm for 20 min. The supernatant was then discarded, the pellet was collected and re-suspended in distilled water and centrifuged again at 10,000 rpm. This process was repeated twice.

### 3.3. The Rate of Nanoparticle Formation

The rate of nanoparticle formation was followed using UV-Vis spectroscopy by monitoring the SPR band of the AgNPs or AuNPs using UV-Vis spectrophotometry. An aliquot (4 mL) of each sample was taken for UV-Vis analysis every 30 min for 18 h in case of AgNPs. For gold nanoparticles, a 4 mL aliquot was taken for UV-Vis analysis every 5 min for 5 h.

### 3.4. Determination of the Total Polyphenolic Content, Reducing Power and the Radical Scavenging Power of the Aqueous Extracts and Fucoidans

The total phenolic content, reducing power and the radical scavenging power of the aqueous extracts of *S. incisifolium* and the fucoidan samples was determined by the method described by Topiwala et al. [36].

### 3.5. Equipment

UV-Vis spectra of all samples were collected using a Cintra UV-Vis spectrophotometer (GBC, Braeside, Victoria, Australia) or a Cary 60 (Varian, Santa Clara, CA, USA) using a 1 cm pathlength quartz cuvette. IR spectra were recorded on a Spectrum 400 FT-IR/FT-NIR spectrophotometer (Perkin Elmer, Waltham, MA, USA) equipped with an ATR accessory. NMR spectra (^1^H and HSQC experiments) were acquired on a 400 MHz Avance IIIHD Nanobay spectrometer (Bruker, Rheinstetten, Germany) equipped with a 5 mm BBO probe at 333 K using standard 1D and 2D NMR pulse sequences. All spectra were referenced to residual undeuterated solvent peaks. The XRD patterns of the lyophilized powder samples of the AgNPs and AuNPs synthesised were acquired on a Bruker AXS (Rheinstetten, Germany) D8 Advance diffractometer (voltage 40 KV; current 40 mA). The XRD spectra were recorded in the range 30–90° using a CuKα (λ = 0.154 nm) monochromatic radiation X-ray source. The size of the NPs was calculated using the Debye-Scherrer equation (Equation (1)) [56].

(1) $$d=\frac{k\lambda }{\beta cos\theta }$$

where *d* is the size; *k* is the Scherrer constant (0.9); λ is the X-ray wavelength; β is the width of XRD peak at half height determined from the graph; θ is the Bragg diffraction angle.

The determination of ion leaching in both AgNP and AuNP was accomplished using an ICap 6200 Inductively Coupled Plasma-Atomic Emission Spectrometer (ICP-AES, Thermo, Waltham, MA, USA). The instrument was calibrated and validated using NIST (National Institute of Standards and Technology, Gaithersburg, MD, USA) traceable standards purchased from Inorganic Ventures (Christiansburg, VA, USA) to quantify selected elements. The solutions were centrifuged at 10000 rpm and the supernatant was collected. From this supernatant, 2 mL aliquot was diluted 10-fold in distilled water. The diluted solution was then submitted for ICP-AES analysis.

Sample morphology and elemental analyses were accomplished using Transmission Electron Microscopy (TEM) and Energy Dispersive X-ray spectroscopy (EDX). TEM images were collected using a Tecnai G2 20 field-emission gun (FEG, FEI, Hillsboro, OR, USA) TEM, operated in bright field mode at an accelerating voltage of 200 kV. EDX spectra were collected using a liquid nitrogen cooled lithium doped silicon detector (EDAX, Hillsboro, OR, USA). The size of nanoparticles in the TEM images was determined using ImageJ software. The hydrodynamic size of nanoparticles was determined using a Zetasizer NanoSeries Instrument (Malvern, Worcestershire, UK). The measurements were done in triplicate and averaged to obtain the mean size of the nanoparticles. Similarly, the zeta potential of the nanoparticles was also determined in triplicate.

### 3.6. Antimicrobial and Cytotoxicity Studies

The nanoparticle reaction mixture was centrifuged at 10,000 rpm for 20 min and the pellet collected and washed several times with Milli-Q water. The pellet was then re-suspended in 3 mL of distilled water. The final concentrations of samples prepared are presented in Table S1.

The selection of microorganisms was carried out such that two gram-positive bacteria *Enterococcus faecalis* (ATCC 51299) and *Staphylococcus aureus* subsp. *aureus* (ATCC 33591), two gram negative strains *Acinetobacter baumannii* (ATCC BAA-1605) and *Klebsiella pneumoniae* subsp. *pneumoniae* (ATCC 700603), and a yeast strain, *Candida albicans* (ATCC 24433) were selected. The agar diffusion assay was carried out according to the method of Dhand et al. [57]. The microorganisms were inoculated into 5 mL growth medium: *A. baumannii* in trypticase soy broth, *E. faecalis* in brain heart infusion broth (supplemented with 4 µg/mL vancomycin), while *K. pneumoniae* subsp. *pneumoniae*, *S. aureus* subsp. *aureus* and *C. albicans* were inoculated into nutrient broth. All strains were incubated at 37 °C, overnight, shaking at 160 rpm on an orbital shaker. The OD_600_ of the cultures were determined and adjusted to OD_600_ = 0.5 for standardization. Each microorganism in liquid suspension (100 µL) was spread onto an agar plate depending on the media it grows on (see above). Four wells were created on each agar plate and 75 µL (refer to Table S1 for concentrations of each sample) of each nanoparticle sample solution was added. Eight replicates, on two separate agar plates (4 replicates per plate), were prepared for each nanoparticle sample. The controls (1 mg/mL) used were vancomycin, ampicillin and chloramphenicol. The plates were then incubated at 37 °C for 24 h. The results were obtained by measuring the diameter of the clear zone (zone of inhibition) around the well.

The cytotoxicity of the synthesised nanoparticles was evaluated against two cancer and one non-cancer cell line. The cell lines MCF-7, MCF-12a, and HT-29 were obtained from ATCC and were grown as follows: MCF-7 and HT-20 cells were grown in Dulbecco’s Modified Eagle’s Media (DMEM) supplemented with 1% of penstrep (penicillin-streptomycin) and 10% foetal bovine serum (FBS). MCF-12a cells were grown in DMEM-F12 to which 1% penstrep and 10% foetal bovine serum were added. In addition to these supplements, 25 µL of hydrocortisone, 10 µL of epidermal growth factor (EGF) and 80 µL of insulin were added to DMEM-F12. All three cell lines were grown under standard culture conditions (37 °C and 5% CO_2_). Cells were trypsinized when they were confluent (70%–90%) after which they were counted on a Countess^TM^ cell counting chamber slide. The number of live cells was used to calculate the volume of cells to be cultured in a 96 well plate. To each well, 100 µL of cells was added (1 × 10^5^ live cells) and incubated for 24 h at 37 °C and 5% CO_2_. The cell viability test was done by using the MTT assay such as was done in the study conducted by Nune et al. [58]. The assay was performed in 96-well microtitre plates. To each well, 100 µL of cells was added (1 × 10^5^ live cells) and incubated for 24 h at 37 °C and 5% CO_2_. The media was discarded after 24 h and the nanoparticles were added in triplicate. Cells and media were used as a control. The 96-well microtitre plate was then incubated for 24 h at 37 °C, 5% CO_2_. The nanoparticles were removed from wells using a multi-channel micropipette after 24 h and the cells were washed with PBS to ensure complete removal of nanoparticles. MTT stock solution (5 mg/mL) 1 mL was mixed with 10 mL of media and 100 µL of the solution was then added to each well. The plate, covered in foil, was incubated for 4 h at 37 °C and 5% CO_2_. After incubation, MTT was then discarded before 100 µL of DMSO (>99.5%) was added to each well and incubated again for 15 min until a purple colour appeared. The absorbance at 570 nm was read on a multi-plate reader (BMG Labtech, Ortenberg, Germany).

The percentage viability was calculated as follows using Equation (2):

(2) $$\%\;\text{viability}=\frac{OD\;of\;test\;sample}{OD\;of\;control}\times 100$$

The cell viability study was done in triplicate and the results were presented as mean ± standard deviation. The *p*-value was calculated with a TTEST function on excel. The *p*-value was represented as *p* < 0.05 with strongest difference and *p* > 0.05 with less difference.

## 4. Conclusions

A “green” method was successfully employed in the synthesis for Au and Ag NPs (at room temperature and pressure conditions) using two differently prepared *S. incisifolium* aqueous extracts (AC and AR extracts). The method of aqueous extract preparation affected the rate at which the NPs were formed as well as their size and shape. NMR and FT-IR spectroscopic studies, as well as DLS and zeta potential measurements, confirmed that complex polysaccharides are the major components of the aqueous extracts, with the latter techniques confirming the presence of these polysaccharides on the surface of the NPs. However, since the pure fucoidans could only produce AgNPs at elevated temperatures, it became apparent that other metabolites present in the aqueous extracts, such as phlorotannins or polyphenols, play an important role in NP formation. The presence of these antioxidants was confirmed by assessment of the antioxidant, reducing power and total polyphenolic ability of the aqueous extracts and fucoidan samples. The method of aqueous extract preparation affected the size, shape and morphology of NPs synthesised. The organic or more non-polar constituents were removed from the AC aqueous extract by prior extraction of the seaweed with organic solvents, followed by the aqueous extraction. This resulted in the production of smaller NPs (which were also produced at a faster rate), as well as NPs which were more uniform in shape and size dispersion. The latter may be explained by the AC extract evidently having a higher polyphenolic content, reducing power and antioxidant activity than the AR extract. The NPs produced using the AR extract resulted in larger, variously shaped NPs. AgNPs and AuNPs control samples were also prepared using widely reported methods of synthesis, i.e., using NaBH_4_ and sodium citrate, respectively.

The preliminary results obtained in this study indicated that the synthesised AgNPs possess potent antimicrobial activities, while the NPs produced using sodium borohydride showed negligible activity against the microorganisms tested. Interestingly, the AR-AgNPs were found to be more effective in killing the microorganisms tested, particularly the gram-negative bacteria. In addition, the methicillin-resistant *S. aureus* and the yeast, *C. albicans*, were the most susceptible to the AgNPs. AuNPs on the other hand, were found to result in much lower zones of inhibition.

The AgNPs synthesised from the aqueous extracts of *S. incisifolium* showed some activity against the HT-29 cell lines, while those synthesised using pure fucoidans showed greater activity against MCF-7. Interestingly, the AgNPs showed lower toxicity levels for the non-cancerous cell line (MCF-12a), showing some selectivity. The concentrations employed in the cytotoxicity studies were much lower than those employed for the antimicrobial studies. The AuNPs, including the SC capped AuNPs, showed little toxicity towards the cancer cell lines. As with the AgNPs, the toxicity of AuNPs towards non-cancer cell lines was negligible compared to that of the cancer cell lines.

## Figures and Tables

**Table 1:** Total polyphenolic content and reducing power of the *S. incisifolium* aqueous extracts, and the fucoidans from *F. vesiculosus* (Fv), *M. pyrifera* (Mp) and *U. pinnatifida* (Up).

Extract	Total Polyphenolic Content (GAE in µg/mg of Dried Seaweed/Fucoidan) *	Total Reducing Power (AAE, in µg/mg of Dried Seaweed/Fucoidan) *
AC	235 ± 0.013	95 ± 0.008
AR	150 ± 0.019	75 ± 0.003
Fv	1 ± 0.0007	10 ± 0.001
Mp	10 ± 0.007	15 ± 0.001
Up	10 ± 0.048	15 ± 0.003

* *p* < 0.05.

**Table 2:** Ag NP data obtained including mean AuNP sizes as determined by TEM, XRD and DLS, as well as Zeta potential measurements and supernatant metal salt percentages.

Sample	TEM Size (nm)		XRD Size (nm)	DLS Size (nm)		Zeta Potential (mV) ^$^	% Metal Salt ^#^
	Mean Size	Range		d_H_	PDI		
SB-AgNP	13.90 ± 9.56	2.04–29.72	25.71	83.65	0.320	−26.4 ± 17.8	0.02
AC-AgNP	22.44 ± 11.85	6.67–53.08	7.29	82.56	0.264	−35.0 ± 4.95	38.2
AR-AgNP	22.94 ± 8.41	3.36–50.99	9.54	76.29	0.568	−40.0 ± 7.62	31.3
Mp-AgNP	20.03 ± 10.97	1.65–46.31	15.28	316.3	0.515	−32.9 ± 3.95	32.1
Fv-AgNP	8.69 ± 3.85	2.35–20.44	- *	126.6	0.326	−44.1 ± 11.0	38.8

* sample quantities obtained were too small for XRD analyses; ^$^ data in triplicate ± standard deviation; ^#^ in supernatant.

**Table 3:** AuNP data obtained including mean AuNP sizes as determined by TEM, XRD and DLS, as well as Zeta potential measurements and supernatant metal salt percentages.

Sample	TEM Size (nm)		XRD Size (nm)	DLS Size (nm)		Zeta Potential (mV) ^$^	% Metal Salt ^#^
	Mean Size	Range		d_H_	PDI		
SC-AuNP	12.38 ± 0.94	3.00–21.38	10.96	37.46	0.158	−56.3 ± 13.9	0.12
AC-AuNP	5.35 ± 3.13	2.17–16.38	22.39	89.62	0.551	−39.3 ± 13.5	14.0
AR-AuNP	66.13 ± 58.30	7.91–268.67	40.12	92.85	0.512	−39.3 ± 14.8	13.6

^$^ data in triplicate ± standard deviation; ^#^ in supernatant.

## Supplementary Materials

Supplementary materials can be accessed at: http://www.mdpi.com/1420-3049/21/12/1633/s1.
