# Supplementary material for: Enhanced Antimicrobial and Anticancer Activity of Silver and Gold Nanoparticles Synthesised Using Sargassum incisifolium Aqueous Extracts
Source: Molecules. 2016 Dec 2;21(12):1633. doi: 10.3390/molecules21121633 (PMC6273965; doi:10.3390/molecules21121633)
Supplement: Supplementary file 1 [file molecules-21-01633-s001.pdf]

Supplementary Materials: Enhanced Antimicrobial and Anticancer Activity of Silver and Gold Nanoparticles Synthesised Using *Sargassum incisifolium* Aqueous Extracts

Mokone Mmola, Marilize Le Roes-Hill, Kim Durrell, John J. Bolton, Nicole Sibuyi, Mervin E. Meyer, Denzil R. Beukes and Edith Antunes

1. Visual and Spectroscopic Characterisation

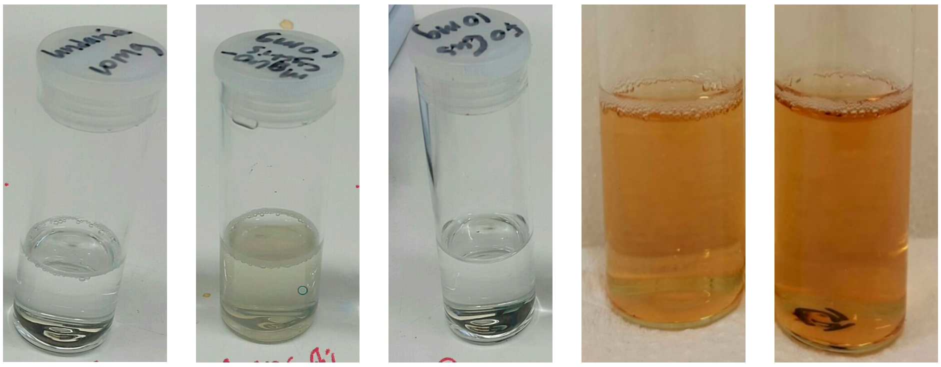

(**a**) (**b**) (**c**) (**d**) (**e**)

Figure S1. Photographs showing the colours of the commercially available fucoidans (**a**) Up; (**b**) Mp; (**c**) Fv, as well as aqueous extracts of *S. incisifolium* (**d**) AC; and (**e**) AR.

1.1. UV-Vis Spectroscopy

UV-Vis spectra of the aqueous extracts and fucoidans in water (Figure S2) revealed the pure fucoidan samples Fucus vesiculosus (Fv) and Undaria pinnatifida (Up) to be featureless, as polysaccaharides are optically transparent. The fucoidans isolated from Macrocystis pyrifera (Mp), while also colourless, displays a broad peak around 250 nm, as do the aqueous extracts (AC and AR), Figure S2, though, the absorption peaks for AC and AR are more pronounced due to the presence of polyphenols. It is clear that the extracts and fucoidans will not mask the observation of the SPR bands for the AgNPs or AuNPs which are expected at ~420 nm and 532 nm, respectively.

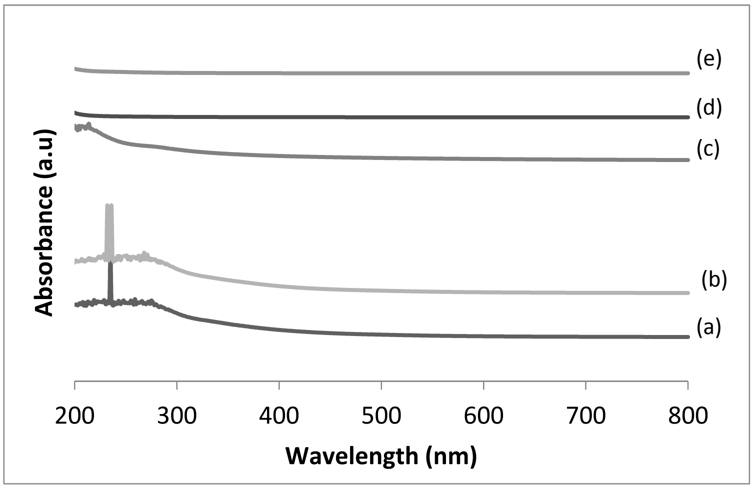

Figure S2. UV-Vis absorption spectra for the *S. incisifolium* aqueous extracts (a) AR and (b) AC; and the commercially available fucoidans (c) Mp; (d) Fv; and (e) Up in water

1.2. NMR Spectroscopy

Further spectroscopic comparison of these extracts and fucoidans was accomplished through ^1H NMR and multiplicity-edited HSQC 2D spectra as shown in Figure S3 (and Figure S4). HSQC spectra provide information on direct ^1H-^{13}C correlations since the large number of overlapping proton signals makes the analysis of the ^1H-NMR spectrum (along the *x*-axis) very difficult. However, using 2D experiments such as HSQC, the carbon signals (along the *y*-dimension) are more 'spread out', providing a significant amount of information. The purpose of this analysis was not to assign the structures of the fucoidans and aqueous extracts, but rather to provide a basis which allows for the identification of distinct structural features for each of the extracts or fucoidans. In addition, it is also possible to distinguish between methyl, methylene and methine signals at a glance. Figure S3 shows an overlay of the HSQC NMR spectra of the *S. incisifolium* aqueous (AC) extract (black), and that of the Fv fucoidan (red). Firstly, a methyl signal at ~δ 18 is clearly apparent, as are the sugar oxymethine carbons between δ 60 and 80. Finally, the anomeric carbons of the sugar moieties are visible around δ 100. Most importantly, an overlay of the two spectra (red and black contours) clearly shows that there are significant differences in carbon and proton signals for the two samples suggesting that the major monosaccharides present in these extracts are quite different. Interestingly, only low intensity signals are present at δ_C/δ_H 102/6.2 (characteristic of phlorotannins/polyphenols) suggesting that phlorotannins are present as minor constituents in the *S. incisifolium* crude extract.

Similar trends were observed when comparing the HSQC spectra of the *S. incisifolium* aqueous extracts and the other fucoidans (Figure S4). Figure S6 shows an overlay of HSQC spectra for all three of the commercially available fucoidans, showing significant overlap of the signals and suggesting a very similar monosaccharide composition for these fucoidans.

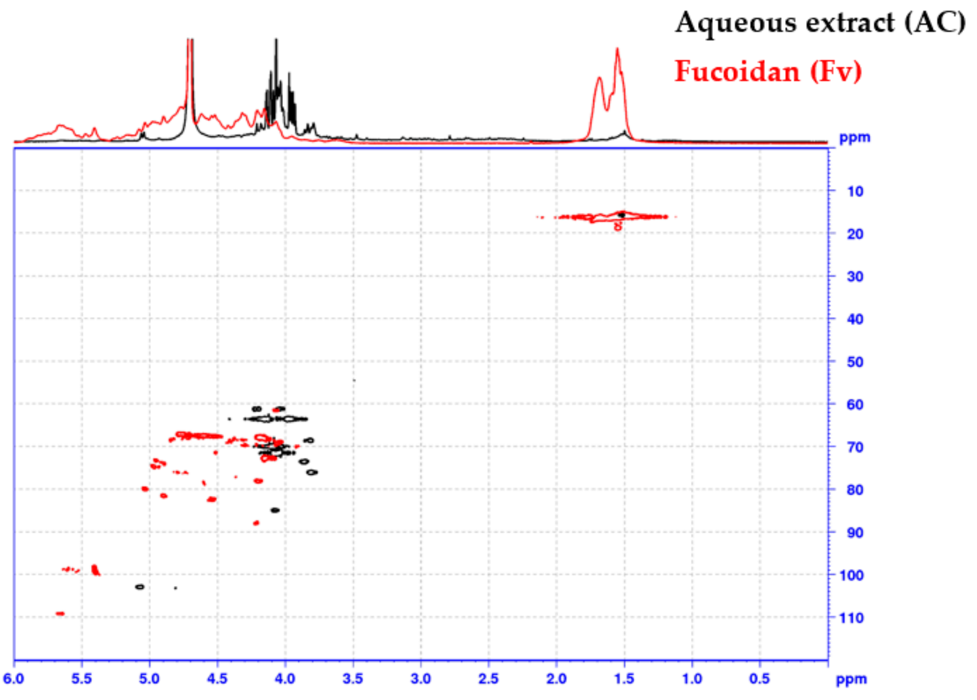

Figure S3. HSQC NMR (400 MHz) spectrum of the *S. incisifolium* AC extract and the Fv fucoidan in D_2O at 333 K.

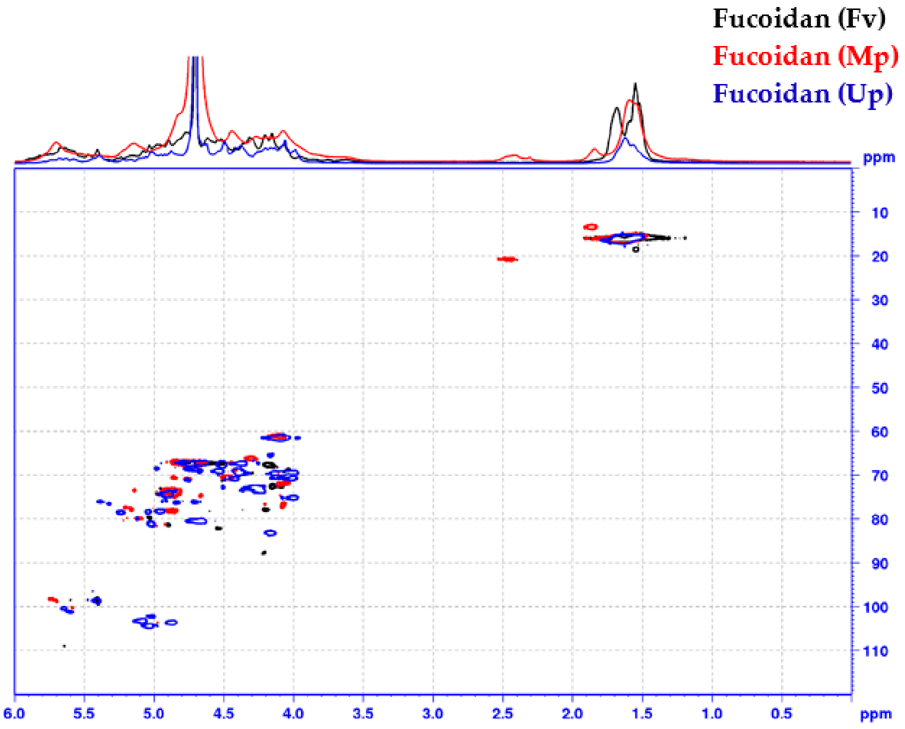

Figure S4. HSQC NMR spectrum of the three fucoidans: Fv (black), Mp (red) and Up (blue). The 400 MHz NMR spectrum was recorded at 333 K in D_2O.

1.3. IR Spectroscopy

FT-IR spectroscopy was also used for comparison of the AC and AR extracts and the fucoidan samples (Figure S5). Key differences in the IR spectra of the fucoidan and the aqueous extract (AR and AC) samples is observed at approximately 800 cm^{-1} which is attributed to the C-O-C group of monosaccharides and is observed in all the spectra except for AC extract. The Mp and Fv fucoidans exhibited a stretch at ~1200 cm^{-1} which may be due to sulphated polysaccharides. All fucoidan samples and aqueous extracts exhibited an OH moiety at around 3200–3400 cm^{-1}, as expected.

FT-IR spectroscopy was used to identify the characteristic peaks associated with the metabolites present in aqueous extract of *S. incisifolium* as well as the pure fucoidans involved in capping the NPs. The FT-IR spectra for AgNP and AuNP are shown in Figures S6 and S7, respectively. Figure S6 shows the C-O-C moiety[4] is still present in all AgNP samples though it has shifted to lower values from ~830 cm^{-1} to approximately 810 cm^{-1}. Furthermore, the peaks observed at approximately 1025 cm^{-1} and 1040 cm^{-1} which are thought to be due to the C-OH of polysaccharides remained largely unchanged. The biggest changes observed were for those peaks observed at approximately 1300 cm^{-1} where peaks that were attributed to the sulfonated polysaccharides were originally present at ~1200 cm^{-1} and a significant decrease in the 1600 cm^{-1} stretch in the AgNPs synthesised. The IR spectra of all the samples all had a broad peak at 3200–3300 cm^{-1} indicating the presence of the OH moieties.

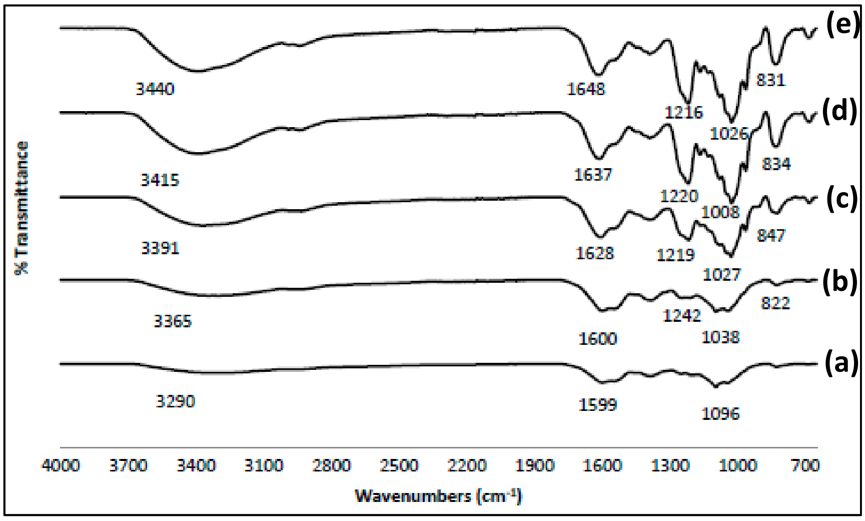

Figure S5. FT-IR spectra of the aqueous extracts of *S. incisifolium* (a) AC; (b) AR and the commercially available fucoidans (c) Mp; (d) Fv and (e) Up

The FT-IR spectra shown in Figure S9 the spectra obtained for AuNPs synthesised. The AC-AuNP and AR-AuNP samples both displayed a peak at 1040 cm^{-1} which is indicative of a C-OH moiety present in polysaccharides. Both AC-AuNP and AR-AuNP contained broad peaks at approximately 3200–3300 cm^{-1} which indicates the presence of a OH group. In addition, the AR-AuNP sample also displayed a peak at 2922 cm^{-1}, attributed to CH stretching which was not clearly observed with the AC-AuNPs.

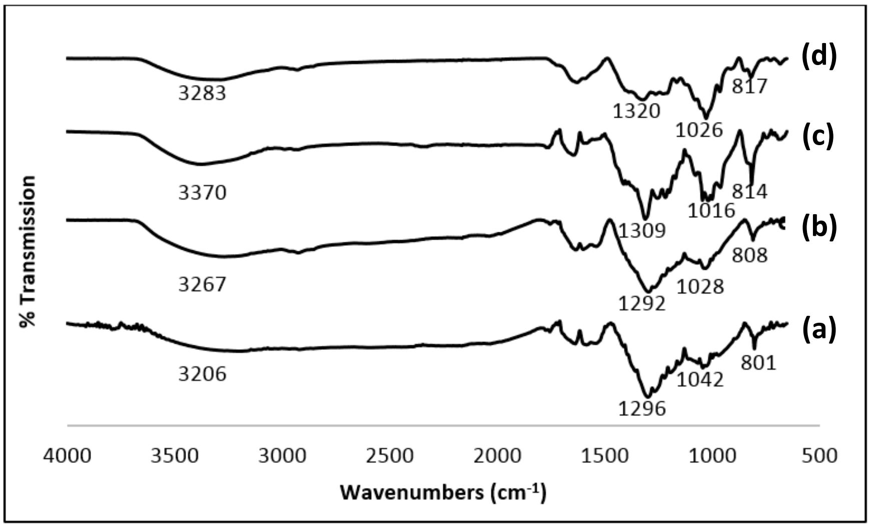

Figure S6. FT-IR spectra obtained for AgNPs synthesised (a) AC-AgNP; (b) AR-AgNP; (c) Fv-AgNP and (d) Mp-AgNP.

Molecules **2016**, *21*, 1633; doi:10.3390/molecules21121633

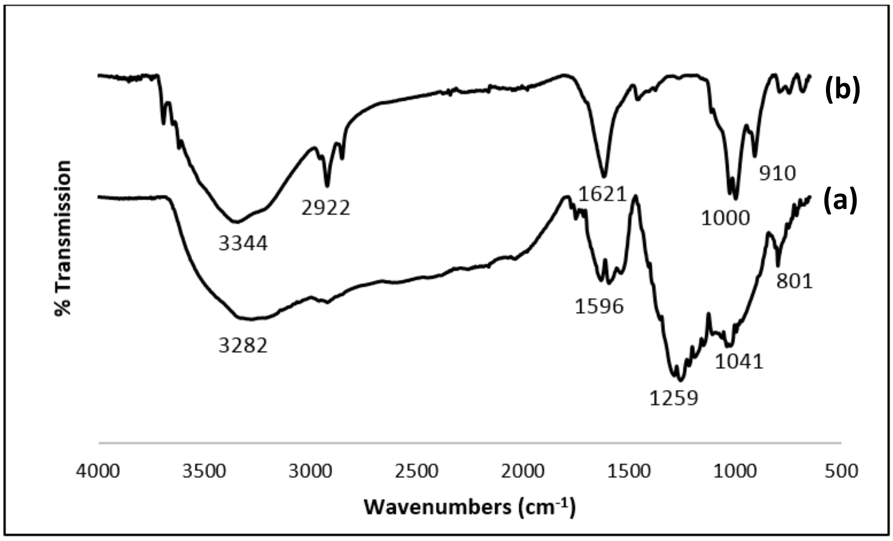

Figure S7. FT-IR spectra of AuNP synthesised to produce the (a) AC-AuNPs and (b) AR-AuNPs.

2. Characterisation of Nanoparticles

2.1. UV-Vis Spectroscopy

2.1.1. Silver Nanoparticles

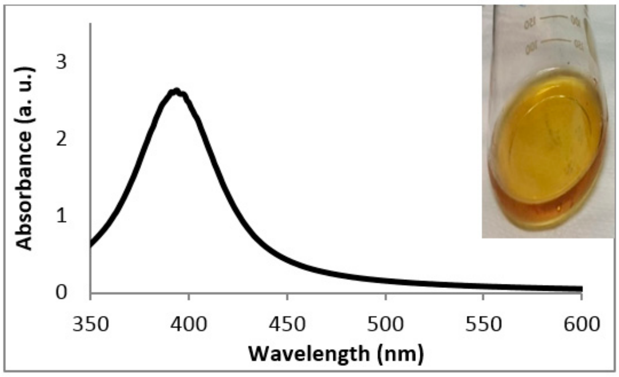

Figure S8. UV-Vis absorbance spectrum of SB-AgNPs and the colour of the solution in water. Inset: Photograph of the colour of the solution after 3 min (in water).

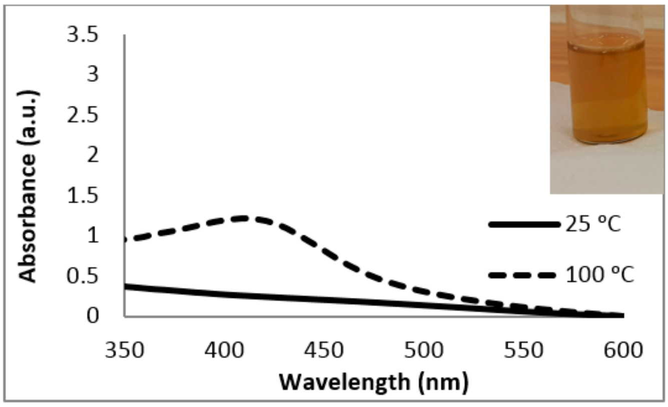

Figure S9. UV-Vis absorption spectra of the Mp-AgNPs after 18 h at room temperature (in water), and after 30 min at 100 °C. Inset: Photograph of the colour of the solution after 30 min (in water).

2.1.2. Gold Nanoparticles

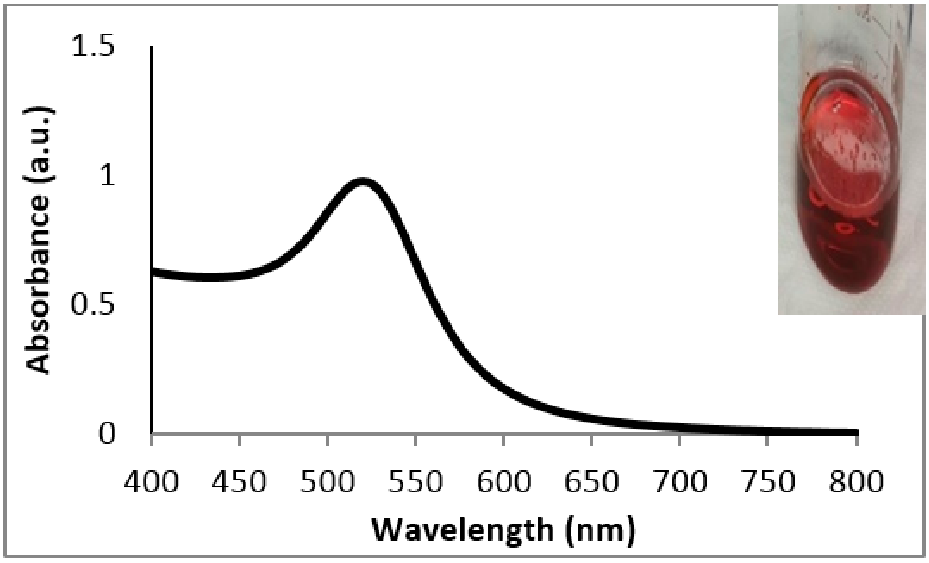

Figure S10. UV-Vis absorption spectrum (in water) of the SC-AuNPs after 10 min at 90 °C. Inset: Photograph of the colour of the solution after 10 min.

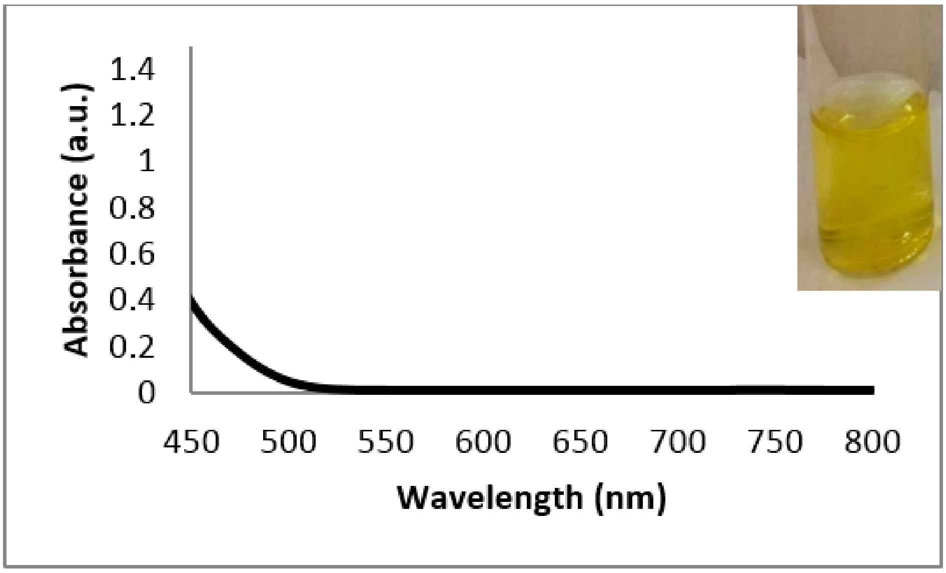

Figure S11. UV-Vis absorption spectrum (in water) of Fv-AuNP after 1 h in water at 100 °C. Inset: Photograph of the colour of the solution after 1 h.

2.2. Transmission Electron Microscopy and Energy Dispersive X-ray Spectroscopy

Elemental analysis of the nanoparticles was accomplished using Energy Dispersive X-ray Spectroscopy (EDX), which was subsequently acquired with the TEM images. Elemental Ag and Au were detected in all samples as expected as shown in Figures S13 and S15. However, traces of other elements which may be part of the sample, sample holder or due to impurities are also observed such as copper, nickel, chlorine and oxygen. Copper and nickel are accounted for since holey carbon coated copper/nickel grids were used in the acquisition of data, while oxygen and chlorine may be due to the starting materials or due to sea salt (NaCl or KCl) which may still be present in the seaweed extract. Oxygen is easily adsorbed onto sample surfaces, which would account for some of its presence.

2.2.1. Silver Nanoparticles

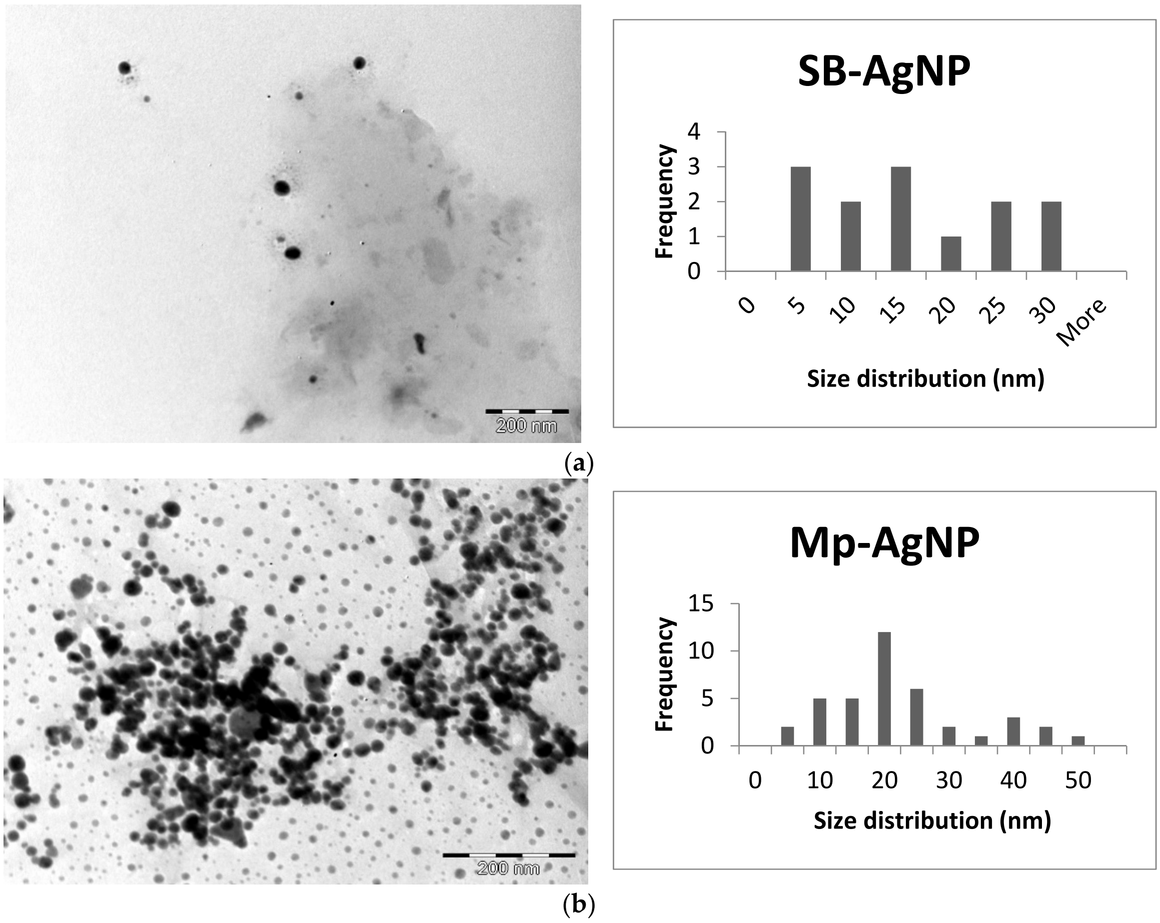

Figure S12. TEM image and NP size distribution obtained for (**a**) the SB-AgNPs and (**b**) Mp-AgNPs.

The EDX spectra (Figure S13) reveal traces of other elements such as oxygen and chlorine which may be due to the starting materials or more likely (because of the manner of sample work up) due to any sea salt (NaCl or KCl) which may still be present in the seaweed extract.

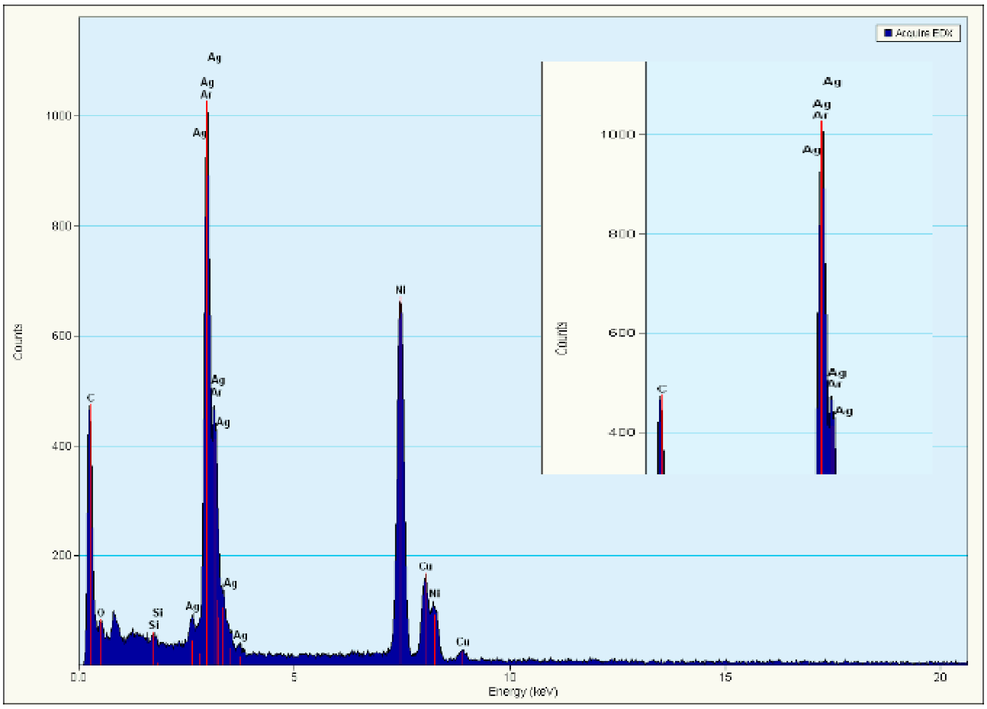

(**a**)

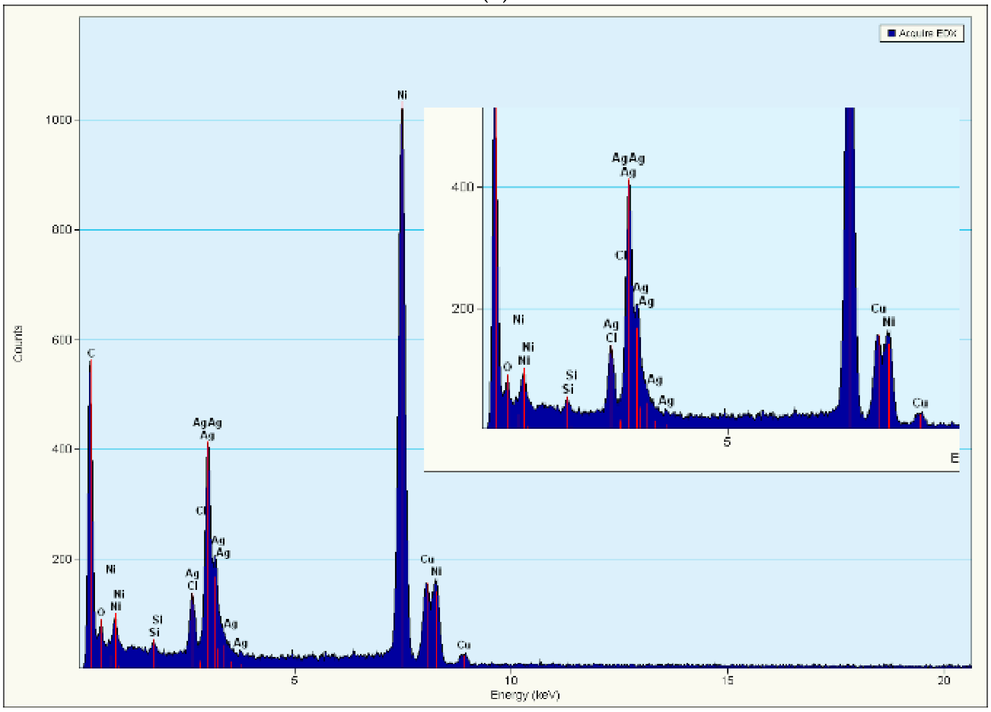

(**b**)

Figure S13. EDX spectra obtained for (**a**) SB-AgNPs and (**b**) AC-AgNPs.

The EDX spectra (Figure S15) reveal traces of other elements such as oxygen and chlorine which may be due to the starting materials or more likely (because of the manner of sample work up) due to any sea salt (NaCl or KCl) which may still be present in the seaweed extract. Inset: enlarged area of EDX spectrum showing Ag peaks.

2.2.2. Gold Nanoparticles

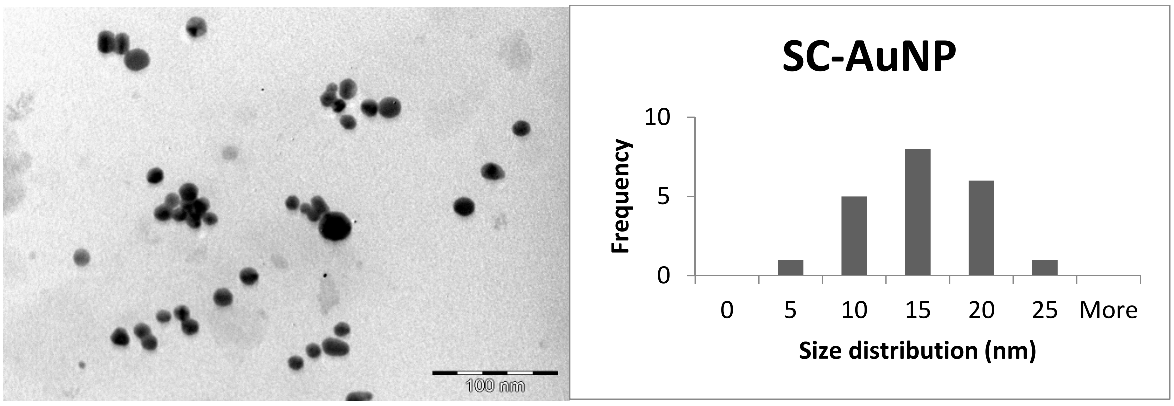

Figure S14. TEM image and NP size distribution obtained for the SC-AuNPs.

The EDX spectra (Figure S15) reveal traces of other elements such as oxygen and chlorine which may be due to the starting materials or more likely (because of the manner of sample work up) due to any sea salt (NaCl or KCl) which may still be present in the seaweed extract.

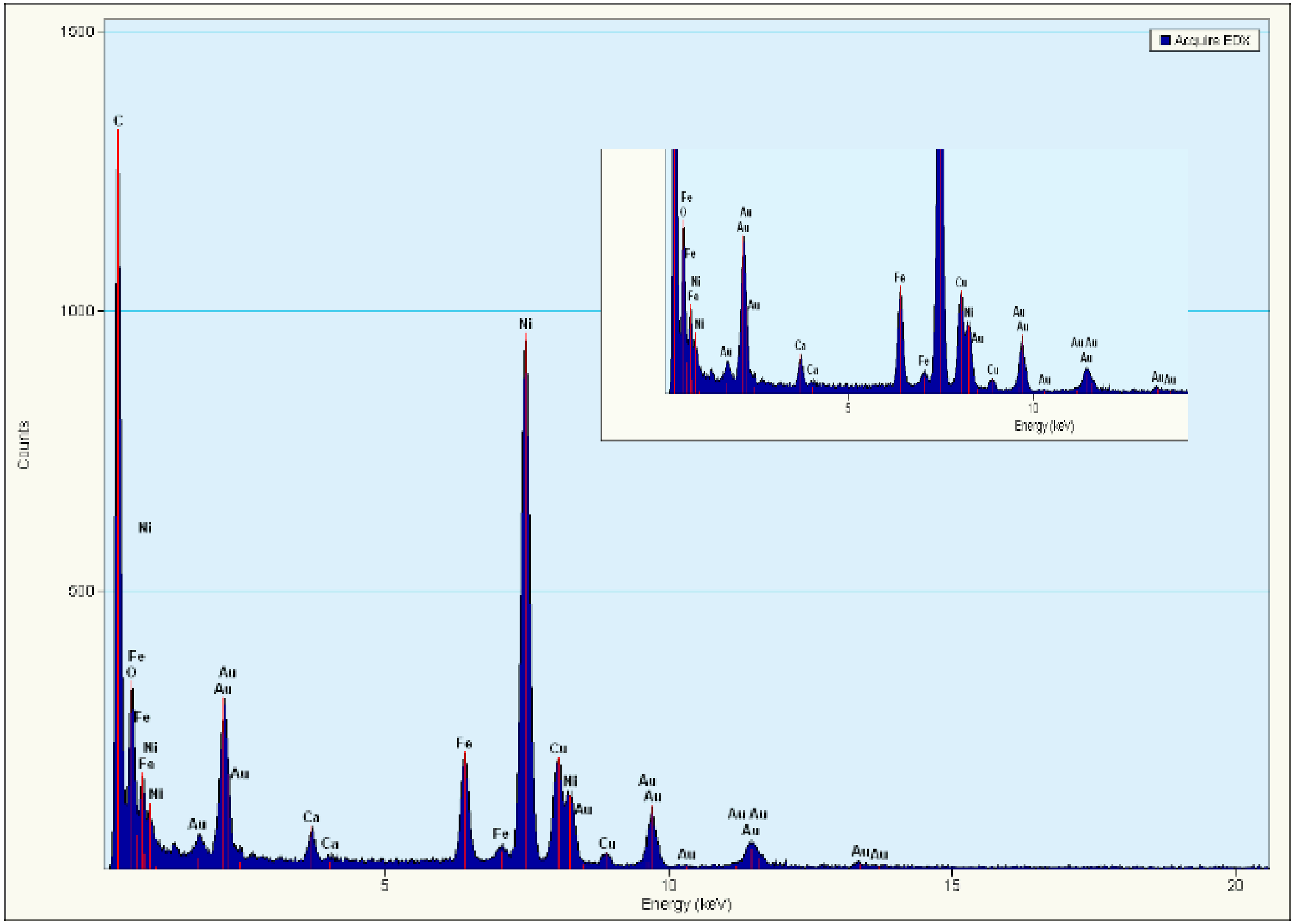

(**a**)

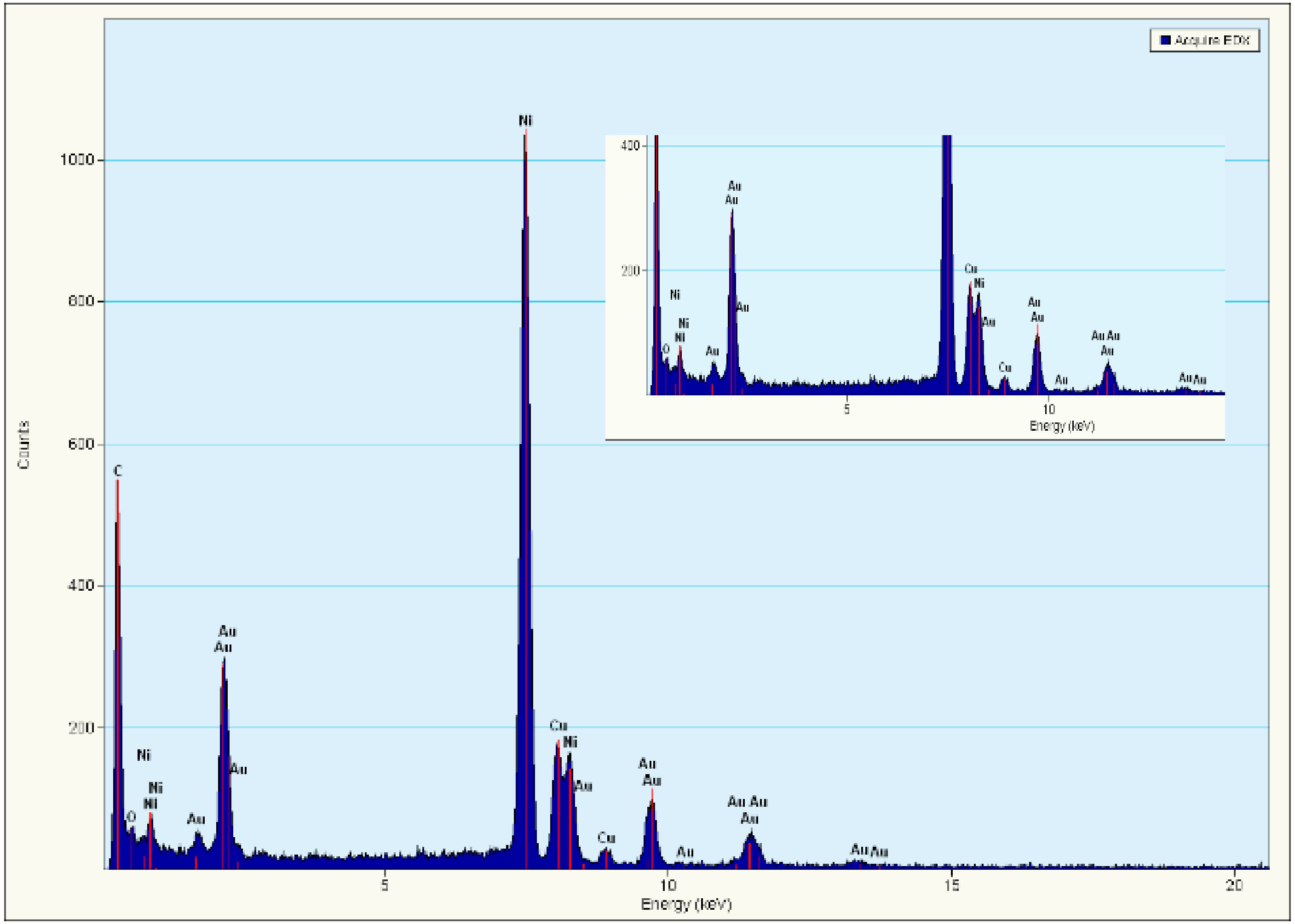

(**b**)

Figure S15. EDX spectra obtained for the (**a**) SC-AuNPs and (**b**) AC-AuNPs. Inset: Zoomed in area showing the Au peaks.

3. Antimicrobial Studies

Table S1. Sample concentrations used for the antimicrobial assay.

Sample Code	Sample Concentration (*Antimicrobial Assay*)
SB-AgNP	0.053 mM
AC-AgNP	0.26 mM
AR-AgNP	0.26 mM
AC-AuNP	0.21 mM
AR-AuNP	0.21 mM
SC-AuNP	0.105 µM
Fv-AgNP	0.26 mM
Fv	0.075 mg/mL
Mp-AgNP	0.26 mM
Mp	0.075 mg/mL
AC	0.015 mg/mL
AR	0.015 mg/mL
Amp	1 mg/mL
Chl	1 mg/mL
Van	1 mg/mL
H_2O	75 µL

Table S2. Antimicrobial activity (well-diffusion assay) of synthesised nanoparticles against a panel of microorganisms.

Sample Code	Ab	Kp	Ef	Sa	Ca
	Zone of Inhibition (Mean ± SD) (mm)				
SB-AgNP	0	0.9 ± 0	0	3.9 ± 0.20	11.5 ± 0.45
AC-AgNP	10.1 ± 0.34	9.4 ± 0.42	8.3 ± 0.62	12.0 ± 0.33	17.5 ± 0.19
AR-AgNP	12.8 ± 0.20	7.5 ± 0.26	7.0 ± 0.19	12.1 ± 0.23	17.8 ± 0.65
AC-AuNP	0	8.0 ± 0.22	0	4.0 ± 0.32	0.5 ± 0
AR-AuNP	0	2.5 ± 0.5	1.5 ± 0	4.0 ± 0.25	0
SC-AuNP	0	0	0	0	0
Fv-AgNP	2.4 ± 0.5	8.6 ± 0.14	3.25 ± 0.29	8.5 ± 0.18	11.3 ± 0.26
Fv	2.8	0	0	0	0
Mp-AgNP	6.3 ± 0.77	9.5 ± 0.19	3.38 ± 0.25	8.8 ± 0.22	15.0 ± 0
Mp	0	0	0	0	0
AC	0	0	0	0	0
AR	0	0	0	0	0
Amp	0	0	0	0	0
Chl	0	18.5 ± 0.25	11.8 ± 0.48	8.0 ± 1.5	0
Van	0	0	0	4.2 ± 0.5	0
H_2O	0	0	0	0	0

Ab = A. baumannii; Kp = K. pneumonia; Ef = E. faicalis; Sa = S. aureus; Ca = C. albicans; SB-AgNP = Silver nanoparticles synthesised using sodium borohydride, AC-AgNP = Silver nanoparticles synthesised using freeze dried aqueous extract with prior organic extraction, AR-AgNP = Silver nanoparticles synthesised from aqueous extract without prior organic extraction, AC-AuNP = Gold nanoparticles synthesised from freeze dried aqueous extract with prior organic extraction, AR-AuNP = Gold nanoparticles synthesised from freeze aqueous extract without prior organic extraction, SC-AuNP =Gold nanoparticles synthesised from sodium cutrate, Fv-AgNP = Silver nanoparticles synthesised from pure fucoidan from *Fucus vesiculosus*, Fv = Pure fucoidan from *Fucus vesiculosus,* Mp-AgNP = Silver nanoparticles synthesised from pure fucoidan from *Macrocystis pyrifera*, Mp = pure fucoidan from *Macrocystis pyrifera*, AC = Freeze dried aqueous extract with prior organic extration, AR = Freeze dried extract without prior organic extraction, Amp = Ampicillin, Chl = Chloramphenicol, Van = Vancomycin.

4. Cytotoxicity Studies

Some issues were encountered with using the MTT dye in these assays. Metallic nanoparticles are known to interfere with MTT absorbance by depleting the free MTT, leading to false positive or negative results. The formazan dye also absorbs in the 500–600 nm range. NPs can cleave the MTT tetrazolium ring and increase light absorption which may influence the final data collected. In our study, it appeared that the nanoparticles themselves did interfere to some extent with the absorbance readings of the formazan dye. Care was thus taken to remove excess NPs before addition of the MTT dye to minimise these effects. To ensure that the AuNPs and AgNPs do not interfere with the absorbance readings, the OD was measured at 570 nm, where the effect of the AuNP SPR band is much lower.

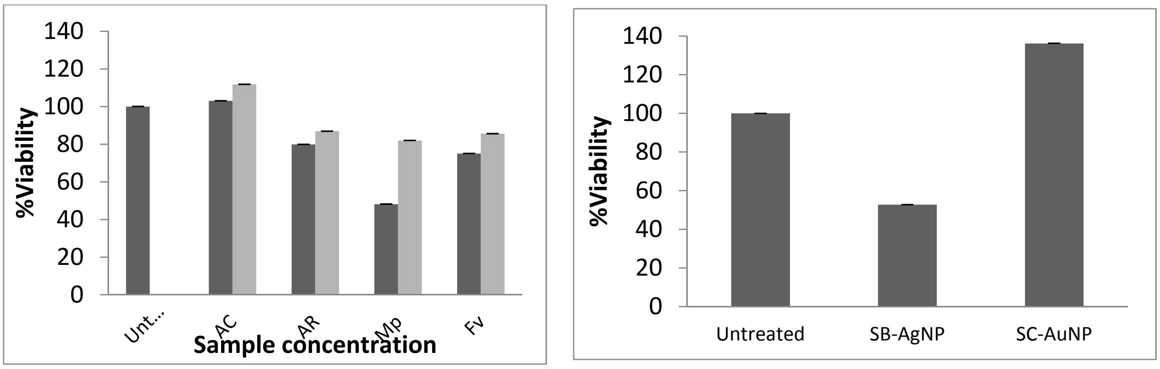

(**a**) (**b**)

Figure S16. Percentage cell viability of the MCF-7 cell line after treatment with (**a**) various agents and (**b**) NPs for 24 h. Concentrations for AC and AR, and Mp and Fv, are the same (first concentration is 0.1 mg/mL and second concentration is 0.05 mg/mL). The concentration of SB-AgNP and SC-AuNP is 0.35 μM and 0.70 μM respectively.

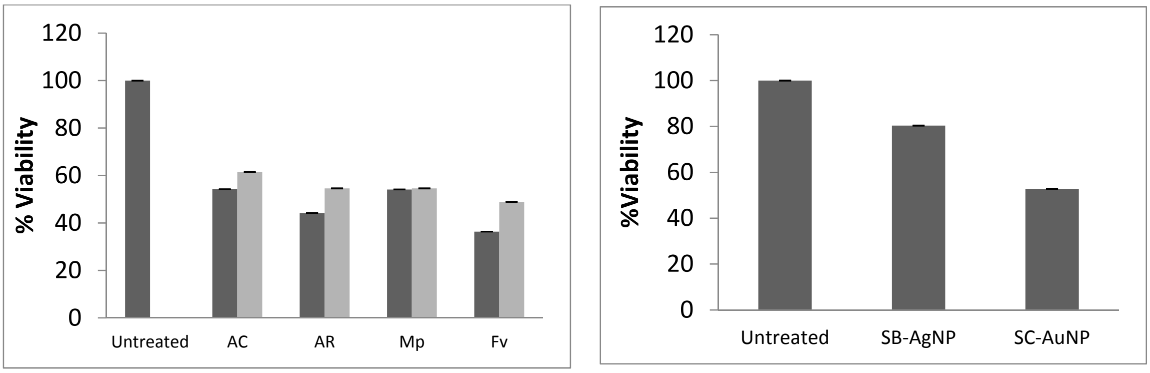

(**a**) (**b**)

Figure S17. Percentage cell viability of the HT-29 cell line after treatment with (**a**) various agents and (**b**) NPs for 24 h. Concentrations for AC and AR are the same (first concentration is 0.1 mg/mL and second concentration is 0.05 mg/mL). The concentrations of Mp and Fv are the same (first concentration is 0.5 mg/mL and the second concentration is 0.25 mg/mL). The concentration of SB-AgNP is 0.35 μM and the concentration of SC-AuNP is 0.70 μM respectively.

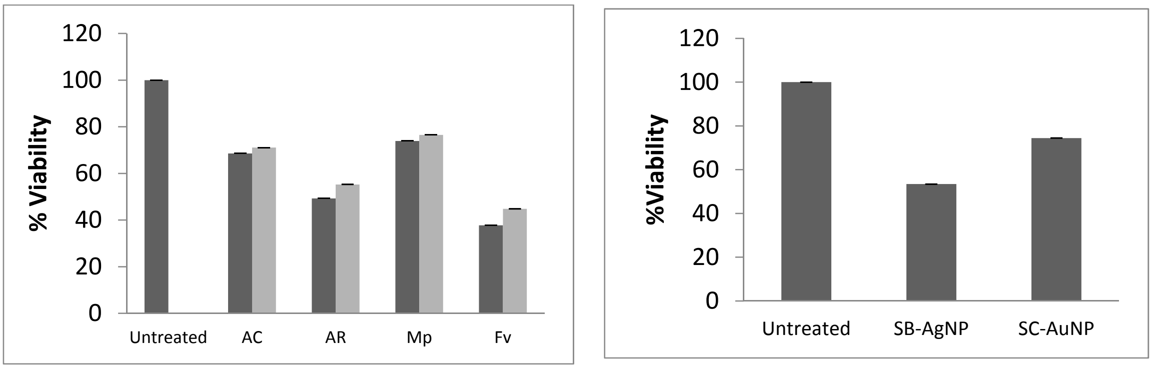

(**a**) (**b**)

Figure S18. After treatment with (**a**) various agents and (**b**) NPs for 24 h. Concentrations for AC and AR are the same (first concentration is 0.1 mg/mL and second concentration is 0.05 mg/mL). The concentrations of Mp and Fv are the same (first concentration is 0.5 mg/mL and the second concentration is 0.25 mg/mL). The concentration of SB-AgNP is 0.35 μM and the concentration of SC-AuNP is 0.70 μM, respectively.
